# Dopaminergic nuclei in the chick midbrain express serotonin receptor subfamily genes

**DOI:** 10.3389/fphys.2022.1030621

**Published:** 2022-11-08

**Authors:** Toshiyuki Fujita, Naoya Aoki, Chihiro Mori, Shouta Serizawa, Fumiko Kihara-Negishi, Koichi J. Homma, Shinji Yamaguchi

**Affiliations:** ^1^ Department of Biological Sciences, Faculty of Pharmaceutical Sciences, Teikyo University, Tokyo, Japan; ^2^ Department of Molecular Biology, Faculty of Pharmaceutical Sciences, Teikyo University, Tokyo, Japan

**Keywords:** dopamine, serotonergic receptor, chick (*Gallus gallus*), A8, substantia nigra pars compacta, ventral tegmental area, optic tectum

## Abstract

Serotonin (5-hydroxytryptamine, 5-HT) is a phylogenetically conserved modulator of numerous aspects of neural functions. Serotonergic neurons in the dorsal and median raphe nucleus provide ascending innervation to the entire forebrain and midbrain. Another important neural modulatory system exists in the midbrain, the dopaminergic system, which is associated to reward processing and motivation control. Dopaminergic neurons are distributed and clustered in the brain, classically designated as groups A8–A16. Among them, groups A8–A10 associated with reward processing and motivation control are located in the midbrain and projected to the forebrain. Recently, midbrain dopaminergic neurons were shown to be innervated by serotonergic neurons and modulated by 5-HT, with the crosstalk between serotonergic and dopaminergic systems attracting increased attention. In birds, previous studies revealed that midbrain dopaminergic neurons are located in the A8-A10 homologous clusters. However, the detailed distribution of dopaminergic neurons and the crosstalk between serotonergic and dopaminergic systems in the bird are poorly understood. To improve the understanding of the regulation of the dopaminergic by the serotonergic system, we performed *in situ* hybridization in the chick brainstem. We prepared RNA probes for chick orthologues of dopaminergic neuron-related genes; tyrosine hydroxylase (*TH*) and dopa decarboxylase (*DDC*), noradrenaline related genes; noradrenaline transporter (*NAT*) and dopamine beta-hydroxylase (*DBH*), and serotonin receptor genes; *5-HTR1A*, *5-HTR1B*, *5-HTR1D*, *5-HTR1E*, *5-HTR1F*, *5-HTR2A*, *5-HTR2B*, *5-HTR2C*, *5-HTR3A*, *5-HTR4*, *5-HTR5A*, and *5-HTR7.* We confirmed that the expression of *tyrosine hydroxylase* (TH) and *NAT* was well matched in all chick dopaminergic nuclei examined. This supported that the compensation of the function of dopamine transporter (DAT) by NAT is a general property of avian dopaminergic neurons. Furthermore, we showed that *5-HTR1A* and *5-HTR1B* were expressed in midbrain dopaminergic nuclei, suggesting the serotonergic regulation of the dopaminergic system *via* these receptors in chicks. Our findings will help us understand the interactions between the dopaminergic and serotonergic systems in birds at the molecular level.

## Introduction

Serotonin (5-hydroxytryptamine, 5-HT) is a phylogenetically conserved modulatory neurotransmitter (Marin et al., 2020). Many studies have suggested that the function of 5-HT is prominent in a broad range of processes, including cognition, behavior, and emotion, with these associations being evolutionarily conserved in the animal kingdom (Kandel et al., 2000; Bacque-Cazenave et al., 2020). For example, 5-HTR1B in the nucleus accumbens is essential for the expression of social reward (Liu et al., 2020) and 5-*HTR1A* knockout mice exhibit elevated anxiety level (O’Leary et al., 2020). Dopamine (DA) is another prominent modulatory neurotransmitter conserved in vertebrates, the functions of which include motor control and motivation-related behavior such as reward and punishment learning (Wise, 2004; Puig et al., 2014). Both the 5-HT and DA systems are involved in processes of motivation-related behavior and interact with each other (Boureau and Dayan, 2011; De Deurwaerdere and Di Giovanni, 2017; Yagishita, 2020; Beyeler et al., 2021; De Deurwaerdere and Di Giovanni, 2021; Peters et al., 2021). To understand the phylogenetic continuity of the neural basis for cognition and emotion, it is necessary to deepen our understanding of the neural circuits that control cognitive and emotional behaviors in animals other than mammals. Birds are excellent model animals to elucidate the evolutionary continuity of the neural basis of cognition and emotion (Rosa Salva et al., 2015; Papini et al., 2019). In birds, the 5-HT system has been suggested to modulate fear-related behavior (Krause et al*.*, 2017; Phi Van et al., 2018; Krause et al., 2019). In addition, the DA system is known to be involved in motor control, learning, and contribution to the acquisition and control of birdsong (Rieke, 1980, Rieke, 1981; Gunturkun, 2005; Fee and Goldberg, 2011). However, the neural circuits that are modulated by 5-HT and DA at a cellular level, and the interactions between the 5-HT and DA systems in avian brains are poorly understood.

In mammals, the dopaminergic neurons are distributed and clustered in the brain, classically designated as cell groups A8-A16 (Dahlstroem and Fuxe, 1964; Bjorklund and Dunnett, 2007). Among them, midbrain dopaminergic neurons cell groups, that is, the retrorubral area (A8), the substantia nigra pars compacta (SNc, A9), and the ventral tegmental area (VTA, A10), have special importance and have been intensively studied in regard to their association with the functions of motor control and motivation-related behavior because they project to a broad area of the forebrain, including the mesolimbic (VTA to nucleus accumbens, amydgala, and hippocampus), mesocortical (VTA to prefrontal cortex), and nigrostriatal (SNc to striatum) projections (Hillarp et al., 1966; Bjorklund and Dunnett, 2007). There is a considerable heterogeneity within and between the nuclei of dopaminergic neurons with respect to multiple aspects, such as connectivity, electrophysiological properties, and molecular and behavioral functions (Lammel et al., 2014; Anderegg et al., 2015; Poulin et al., 2020). In addition, the VTA contains diverse populations of dopaminergic, glutamatergic, and GABAergic neurons, as well as combinatorial neurons that release more than one neurotransmitter and a complex microcircuitry that integrates interactions among local dopaminergic, glutamatergic, and GABAergic neurons (Morales and Margolis, 2017). The midbrain dopaminergic nuclei with such characteristics have been known to receive serotonergic neuron projections from the dorsal raphe (DR) nucleus (Ogawa et al., 2014; Beier et al., 2015; Ogawa and Watabe-Uchida, 2018). The type of serotonergic receptors expressed in dopaminergic neurons and the mechanism by which they are regulated in neural circuit levels has become clear in recent years (Wang et al., 2019; Peters et al., 2021).

In birds, previous studies have revealed that the midbrain dopaminergic neurons are located in the A8–A10 homologous clusters in the midbrain (Ikeda and Goto, 1971; Dube and Parent, 1981; Guglielmone and Panzica, 1984; Moons et al., 1994; Reiner et al., 2004). Moreover, the afferent and efferent connections of the midbrain dopaminergic nuclei in birds are similar in extent to those of mammals (Csillag, 1999; Durstewitz et al., 1999; Mezey and Csillag, 2002; Balint and Csillag, 2007; Balint et al., 2011). In terms of molecular characteristics, the dopaminergic system of birds was also thought to be largely conserved with that of mammals (Yamamoto Vernier, 2011). However, in recent years, sauropsids, including birds and reptiles, were reported to have lost the dopamine transporter (*DAT*) gene from the genome, with the noradrenaline transporter (NAT) being proposed to compensate for the function of DAT in sauropsids (Lovell et al., 2015). Therefore, the degree of conservation of the bird dopaminergic system with that of the mammalian dopaminergic system needs to be reexamined. Furthermore, in avian brains, the interactions between the dopaminergic and serotonergic systems are still poorly understood.

In the present study, to better understand the midbrain dopamine system in birds and to improve our understanding of the interactions between the dopamine and serotonin systems in birds, we investigated the chick brainstem at the molecular level using *in situ* hybridization (ISH). We selected chick orthologs of dopaminergic neuron-related marker genes: *tyrosine hydroxylase* (*TH*) and dopa *decarboxylase* (*DDC*); noradrenaline neuron-related marker genes: *noradrenaline transporter* (*NAT*) and *dopamine beta-hydroxylase* (*DBH*); and serotonin receptor genes: *5-HTR1A*, *5-HTR1B*, *5-HTR1D*, *5-HTR1E*, *5-HTR1F*, *5-HTR2A*, *5-HTR2B*, *5-HTR2C*, *5-HTR3A*, *5-HTR4*, *5-HTR5A*, and *5-HTR7.* We confirmed that the expression of *TH* and *NAT* was well matched in all chick dopaminergic nuclei examined. This supported that the compensation of the function of DAT by NAT is a general property of avian dopaminergic neurons. Furthermore, we found that *5-HTR1A* and *5-HTR1B* were expressed in dopaminergic nuclei and in cells other than dopaminergic neurons. Understanding the nature of serotonin receptor-expressing cells in avian dopaminergic nuclei will help us understand the serotonergic regulation of the avian dopamine system and elucidate the functional correspondences between the avian and mammalian dopamine systems.

## Materials and methods

### Animals

Fertilized eggs of domestic chickens of the Cobb strain (*Gallus gallus domesticus*) were purchased from a local company (3-M, Aichi, Japan). Eggs were incubated at the Teikyo University (Kaga, Itabashi-ku, Tokyo, Japan). Animal experiments were performed as described previously by Yamaguchi et al. (2008a, 2008b). Briefly, newly hatched chicks (P0) were transferred to dark plastic enclosures in a dark warm cage at 30°C for 1 d (P1). We used 10 chick brainstems for the detection of *tyrosine hydroxylase* (*TH*), 8 for *noradrenaline transporter* (*NAT)*, 7 for *dopa decarboxylase* (*DDC*), 7 for dopamine beta-hydroxylase (*DBH*), 5 for 5-*HTR1A*, 5 for 5-*HTR1B*, 3 for 5-*HTR1D*, 3 for 5-*HTR1E*, 3 for 5-*HTR1F*, 4 for 5-*HTR2A*, 3 for 5-*HTR2B*, 4 for 5-*HTR2C*, 4 for 5-*HTR3A*, 3 for 5-*HTR4*, 3 for 5-*HTR5A*, 3 for 5-*HTR7,* and 2 for reverse-transcription polymerase chain reaction (RT-PCR) experiments (Supplementary Table S1). Previous studies showed that embryonic development of the catecholamine system is almost completed before hatching (Guglielmone and Panzica, 1984) and distribution of dopamine neurons does not show difference between ages after hatched (Moons et al., 1994), suggesting that the distribution of dopamine neurons in the brainstem of P1 chick was almost same as that in the adult chick. The expression level of 5-HTRs may change during post-natal development, but this is the issue needs to be addressed in the future. All experimental procedures were reviewed and approved by the Committee on Animal Experiments of Teikyo University and conducted in accordance with the guidelines of the national regulations for animal welfare in Japan.

### Histology preparations

First, P1 chicks were anesthetized by an intraperitoneal injection (0.40 ml/individual) of a 1:1 mixture of ketamine (10 mg/ml, Ketalar-10, Sankyo Co., Tokyo, Japan) and xylazine (2 mg/ml, Sigma, St. Louis, MO, United States). Then, anesthetized chicks were transcardially perfused with 4% paraformaldehyde in 0.1 M phosphate buffered saline (pH 7.5, PFA-PBS). After perfusion, whole brains were quickly dissected up to the spinal cord level, immediately immersed in PFA-PBS for 1 day at 4°C and placed in an 18% sucrose/PFA-PBS solution for cryoprotection for 2 day at 4°C. Next, brains with sucrose substitution were embedded in Tissue-Tek OCT compound (Sakura Finetechnical, Tokyo, Japan), frozen promptly on dry ice, and stored at −80°C until sectioning. Frozen brain blocks were cut into 18 µm-thick sections using a cryostat (Leica CM3050S or Leica CM 1850, Leica Biosystems, Nußloch, Germany). We mounted serial sections on glass slide (Platinum PRO, cat# PRO-01, MATSUNAMI Glass Ind., Osaka, Japan). The level of serial coronal sections (A3.4 to A1.0) corresponded to those of the atlas by Kuenzel and Masson (1988).

### cDNA cloning and RNA probe preparations

Total RNA was extracted from the chick brain using the TRIzol reagent (Invitrogen, Carlsbad, CA, United States) and reverse-transcribed using the SuperScript III kit (Invitrogen) with oligo (dT) primers, according to the manufacturer’s instructions. RT-PCR was performed using the following gene-specific primer (forward and reverse) pairs: *TH:* 5′-GCC​TCA​TTG​AAG​ATG​CCA​GG-3′ and 5′-AGC​CAG​TCC​TCT​CTT​TCA​GG-3′, respectively; *NAT:* 5′-GAA​CAA​ACA​GAT​CCA​GCC​CG-3′ and 5′-CTT​CAC​GCC​TTT​CCA​CAG​AC-3′, respectively; *DDC:* 5′-TGG​TGG​ACT​ACG​TTG​CAG​AT-3′ and 5′-TTG​TCA​AAG​GAG​CAG​CAA​GG-3′, respectively; *DBH:* 5′-AGC​CAA​GAG​ACG​ACC​TAC​TG-3′ and 5′-CAT​TGC​TCC​TGT​TCT​CCG​TG-3′, respectively. PCR amplicons were subcloned into the pGEM-T easy vector (Promega, Madison, WI, United States). All sequences were confirmed using Sanger sequencing. For 5-*HTR1A, 5-HTR1B, 5-HTR1D, 5-HTR1E, 5-HTR1F, 5-HTR2A, 5-HTR2B, 5-HTR2C, 5-HTR3A, 5-HTR4, 5-HTR5A,* and 5-*HTR7* probes, we used previously constructed plasmids (Fujita et al., 2020; Fujita et al., 2022a) (Supplementary Table S2). Plasmids containing the cDNA fragments for *TH, NAT, DDC, DBH, 5-HTR1A, 5-HTR1B, 5-HTR1D, 5-HTR1E, 5-HTR1F, 5-HTR2A, 5-HTR2B, 5-HTR2C, 5-HTR3A, 5-HTR4, 5-HTR5A*, and 5-*HTR7* were amplified by PCR using the M13 primer pair. Amplicons containing the T7 and SP6 promoter sites were purified using a PCR purification kit (Qiagen, Valencia, CA, United States). Digoxigenin (DIG)-labeled sense and antisense RNA probes were prepared by *in vitro* transcription using a DIG RNA labeling kit (Roche, Basel, Switzerland). All subclones of *5-HTR*s were confirmed by Sanger sequencing (Supplementary Figure S1) and were validated by denaturing RNA electrophoresis using agarose gels (Supplementary Figure S2). For double ISH analysis, *TH* fluorescein-labeled sense and antisense RNA probes were prepared using a fluorescein RNA labeling kit (Roche) in the same manner as above.

### *In situ* hybridization

ISH experiments were performed as described previously by Fujita et al. (2019), with some modifications. Briefly, brain sections on slide glasses were refixed in 4% PFA-PBS, pretreated, and hybridized with DIG-labeled RNA probes at 70°C. After stringent washes, hybridized probes were detected *via* an immunohistochemical examination with an alkaline phosphatase-conjugated anti-DIG antibody (1:1000; Roche). For signal visualization, a chromogenic reaction with a nitro blue tetrazolium/5-bromo-4-chloro-3-indolyl phosphate (NBT/BCIP) was performed at 25°C for the following durations: *TH, NAT, DDC,* and *DBH,* 15–19 h; *5-HTR1A*, *5-HTR1B*, *5-HTR1D,* and *5-HTR1E*, 18.25–18.5 h; *5-HTR2A, 5-HTR2C,* and *5-HTR3A*, 18.25–18.6 h; 5-*HTR1F*, 18.25–20 h; 5-*HTR2B* and *5-HTR5A*, 20 h; 5-*HTR4*, 18.5–18.6 h; and 5-*HTR7*, 18.5 h. Sense probes were used as negative controls in every experiment.

### Double *in situ* hybridization

Double ISH experiments were performed as described previously by Fujita et al. (2022a). Briefly, during hybridization, DIG-labeled and fluorescein-labeled RNA probes were mixed and hybridized simultaneously. After the first chromogenic reaction with NBT/BCIP, sections on slide glasses were treated with 100 mM glycine (pH 2.2) for antibody detachment. After washing in PBS, fluorescein-labeled probes were detected immunohistochemically using an alkaline phosphatase-conjugated anti-fluorescein antibody (1:1000; Roche). For signal visualization, the second color chromogenic reactions were performed at 25°C using SIGMAFAST Fast Red TR/Naphthol AS-MX tablets (cat#F4523, Sigma-Aldrich, St. Louis, MO, United States). The durations for the first and second reactions were as follows: first reaction, *5-HTR1A* or *5-HTR1B*, 2–3 h and second reaction, *TH*, 18–114 h. Sense probes were used as negative controls in every experiment. We only determined clearly double labelled cells as double labelled.

### Imaging and data processing

Digital photographs of sections on each slide glass were acquired semiautomatically using NanoZoomer 2.0 HT or NanoZoomer XR systems (Hamamatsu Photonics, Shizuoka, Japan). The microscopic fields of interest were cropped using the NDP. view2 software (ver. 2.7.25, https://www.hamamatsu.com/; Hamamatsu Photonics, Shizuoka, Japan). For ISH imaging, cropped images were converted to 8-bit images, and their brightness and contrast were adjusted using ImageJ (ver. 1.52a, https://imagej.nih.gov/ij/; National Institute of Health, Bethesda, MD, United States). For fluorescent imaging, digital photographs were acquired semiautomatically using the NanoZoomer XR system in the bright and fluorescent modes using a TRITC filter.

## Results

### Selection of chick orthologues of mammalian dopaminergic and noradrenergic neuron-related genes and 5-HTR genes

To clarify the distribution of DA neurons and the crosstalk between the dopaminergic and serotonergic system in the chick midbrain, we selected chick orthologs of the following mammalian dopaminergic neuron-related genes: *TH* and *DDC*, and noradrenergic neuron-related genes: *NAT* and *DBH,* as well as all chick *5-HTR* genes (*5-HTR1A, 5-HTR1B, 5-HTR1D, 5-HTR1E, 5-HTR1F, 5-HTR2A, 5-HTR2B, 5-HTR2C, 5-HTR3A, 5-HTR4, 5-HTR5A*, and *5-HTR7*; except *5-HTR6*, for which we could not obtain a PCR amplicon). The features of dopaminergic and noradrenergic neuron-related genes are as follows: TH is a rate-limiting enzyme for dopamine biosynthesis; DDC is an enzyme necessary for the stepwise production of dopamine, but also involved in a wider range of processes, including serotonin production; NAT is a transporter for noradrenaline reuptake from the synaptic cleft to the neuron that released noradrenaline and is encoded by the *solute carrier family 6 member 2* (*Slc6a2*) gene; and DBH is an enzyme responsible for noradrenaline production from dopamine (Bjorklund and Dunnett, 2007; Yamamoto and Vernier, 2011). We found that orthologs between chicks and humans exhibited the following sequence similarities (protein and DNA): *TH*, 77.2% and 74.8%, respectively; *DDC*, 77.4% and 73.5%, respectively; *NAT*, 85.7% and 76.1%, respectively; *DBH*, 69% and 73.1%, respectively. We also searched for sequence similarities between *5-HTR* genes and genes in other animals in previous studies (Fujita et al., 2020; Fujita et al., 2022a). The information summary of these ortholog gene probes is provided in Table 1 and Supplementary Table S2. We designed probes to detect multiple transcript variants of the orthologs registered in the database. We also performed *in situ* hybridization (ISH) and analyzed the expression patterns of orthologs in the brainstem of chicks.

**TABLE 1:** Overview of dopaminergic and noradrenergic neuron-related gene probes used in this study.

Accession number	Gene symbol	Molecular characteristics
NM_204805.2	TH	Enzyme
XM_419032.7	DDC	Enzyme
NM_204716.2	NAT	Membrane transporter
XM_040686111.1	DBH	Enzyme

DBH, dopamine beta-hydroxylase; DDC, dopa decarboxylase; NAT, noradrenaline transporter; TH, tyrosine hydroxylase.

### Expression of tyrosine hydroxylase*,* dopa decarboxylase*,* noradrenaline transporter*,* and dopamine beta-hydroxylase in the chick brainstem

**FIGURE 1:**
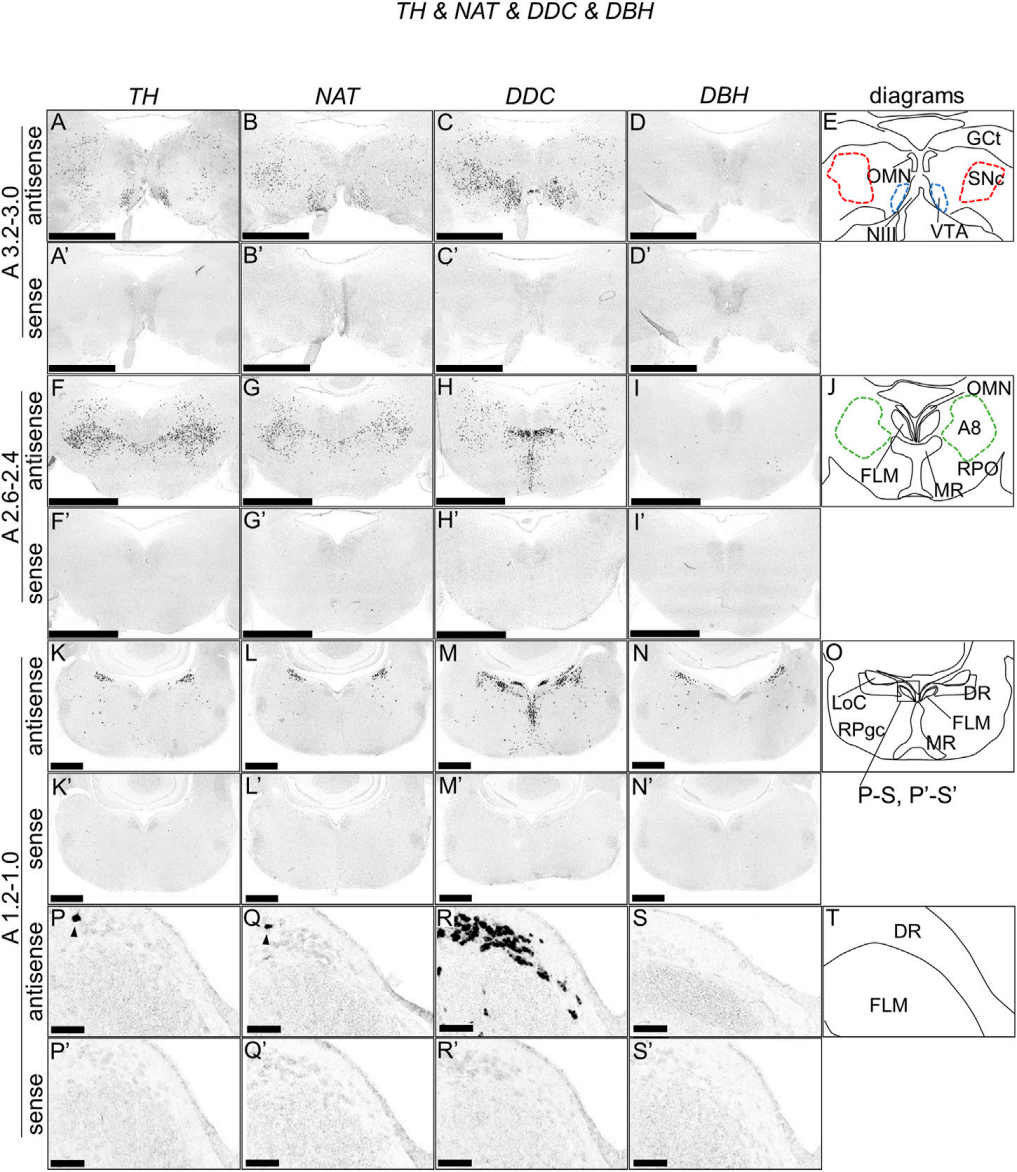
*In situ* hybridization of *TH, NAT*, *DDC*, and *DBH* in the P1 chick brainstem. Digoxigenin-labeled RNA antisense [*TH*, **(A,F,K,P)**, *NAT*, **(B,G,L,Q)**, *DDC*, **(C,H,M,R)**, and *DBH*, **(D,I,N,S)**] and sense [*TH*, **(A′,F′,K′,P′)**, *NAT*, **(B′,G′,L′,Q′)**, *DDC*, **(C′,H′,M′,R′)**, and *DBH*, **(D′,I′,N′,S′)**] probes were used for *in situ* hybridization in coronal sections of P1 chick brainstems. To evaluate the expression patterns of *TH, NAT, DDC,* and *DBH*, sections of 10 P1 chick brainstems were analyzed for *TH*, 8 for *NAT*, 7 for *DDC,* and 7 for *DBH.* Representative images of the levels of expression in neighboring sections (A3.2–3.0, A2.6–2.4, and A1.2–1.0) from 3 P1 chick brainstems. **(P–S** and **P′–S′)** Magnified views of brainstem areas in the box in **(O)**. The levels of expression in these sections were in accordance with those mentioned in the Kuenzel and Masson’s chick atlas (Kuenzel and Masson, 1988). **(E,J,O,T)** Diagrams of coronal sections shown in panels **(A), (F), (K),** and **(P)**. Arrowheads indicate signals. A8: A8 cell group; DR, dorsal raphe; FLM, fasciculus longitudinalis medialis; GCt, griseum centrale; LoC, locus coeruleus; MR, median raphe; NIII, nervus oculomotorius; OMN, oculomotor nucleus; P1, post-hatch day 1; RPgc, nucleus reticularis pontis caudalis; RPO, nucleus reticularis pontis oralis; VTA, ventral tegmental area. Scale bars = 2.5 mm **(A–I)** and **(A′–I′)**, 1 mm **(K–N)** and **(K′–N′)**, and 100 µm **(P–S)** and **(P′–S′)**.

We performed ISH analysis to reveal the expression patterns of the dopaminergic and noradrenergic neuron-related genes, *TH, DDC, NAT*, and *DBH* in neighboring sections around A3.4 to A 1.0 in the brainstems of naive chicks on post-hatch day 1 (P1). Accordingly, we detected cells with strong signals of *TH* and *NAT* in the VTA, SNc, GCt, A8, MR, LoC, RPgc, and DR (Figures 1A,B,F,G,K,L,P,Q, Figures 1A′,B′,F′,G′,K′,L′,P′,Q′). We also observed cells with strong signals of *DDC* in the VTA, SNc, between the VTA and SNc, A8, MR, LoC, and DR (Figures 1C,H,M,R, Figures 1C′,H′,M′,R′). We also found cells with strong signals of *DBH* in between the A8 and MR, LoC, and between the LoC and RPgc (Figures 1D,I,N, Figures 1D′,I′,N′). As noradrenergic neurons contain dopamine as a synthetic intermediate, noradrenergic neurons express *TH, DDC*, and *DBH*, whereas dopaminergic neurons express only *TH* and *DDC* but not *DBH* (Bjorklund and Dunnett, 2007; Yamamoto and Vernier, 2011). In mammals, *DAT* is considered to be the most accurate single-gene marker gene for dopaminergic neurons (Lemmel et al., 2015). However, in amniotes, including birds, *DAT* is deleted from the genome, with NAT being suggested to compensate the function of DAT (Lovell et al., 2015). We found that the expression patterns of *TH, NAT*, and *DDC* were similar in the SNc, VTA, and A8 in chicks, and further confirmed the absence of *DBH* signals.

### Expression of *5-HTR1A* in the chick brainstem

**FIGURE 2:**
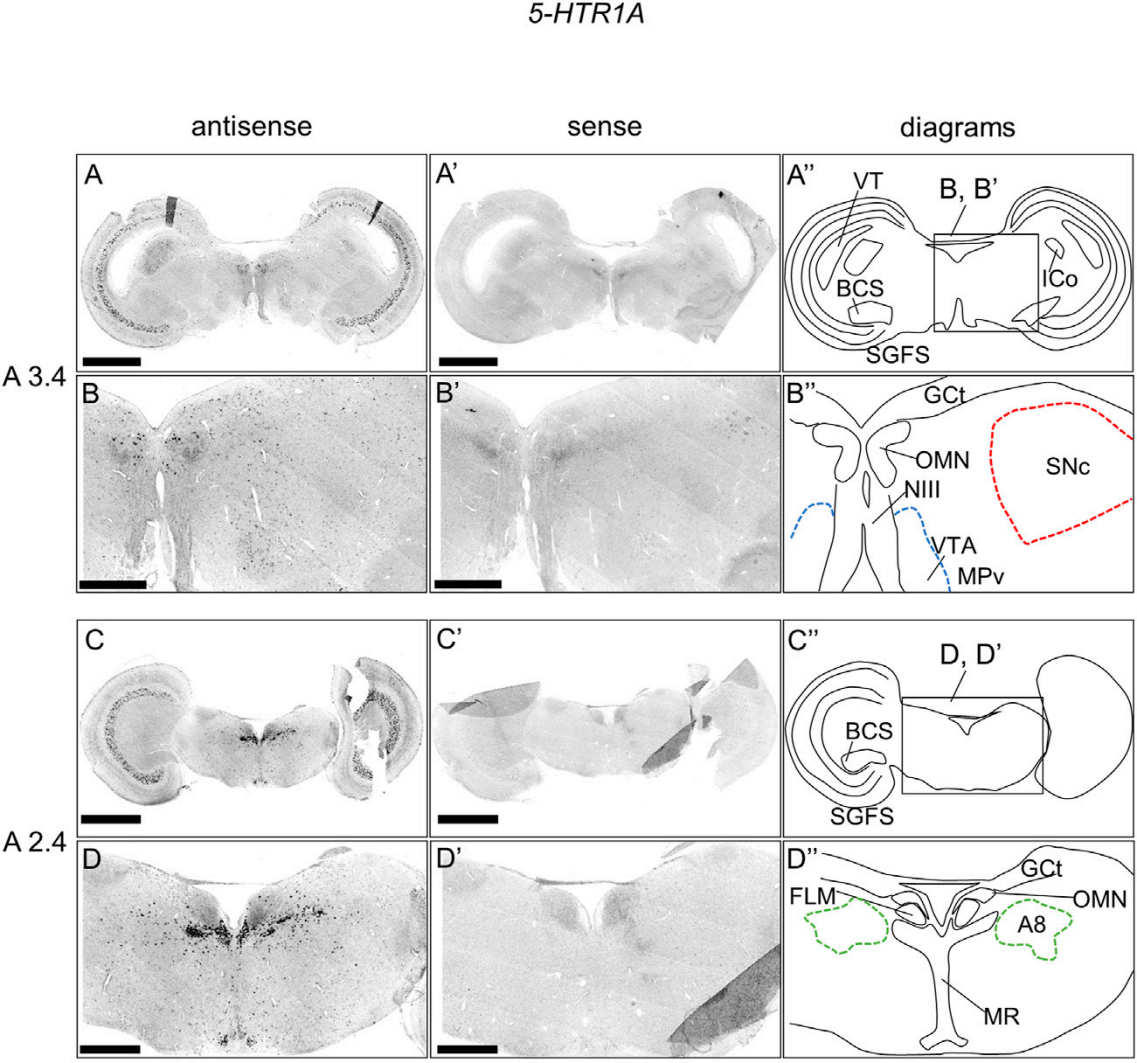
*In situ* hybridization of *5-HTR1A* in the P1 chick brainstem. Digoxigenin-labeled RNA antisense **(A–D)** and sense **(A′–D′)** *5-HTR1A* probes were used for *in situ* hybridization in coronal sections of P1 chick brainstems. To evaluate the expression patterns of *5-HTR1A*, we analyzed sections from five chick brainstems; representative images of chick brainstem sections are shown. **(B)** and **(B′)** and **(D)** and **(D′)** Magnified views of brain areas in the box in **(A′′)** and **(C′′)**. **(A′′–D′′)** Diagrams of coronal sections shown in the rightmost panels. The levels of expression in sections (A 3.4 and A 2.4) were in accordance with those mentioned in the Kuenzel and Masson’s chick atlas (Kuenzel and Masson, 1988). A8: A8 cell group; BCS, brachium colliculi superiors; FLM, fasciculus longitudinalis medialis; GCt, griseum centrale; ICo, nucleus intercolicularis; MR, median raphe; NIII, nervus oculomotorius; OMN, oculomotor nucleus; P1, post-hatch day 1; PPT, pedunculopontine tegmental nucleus; SGFS, stratum griseum et fibrosum superficiale; SNc, substantia nigra pars compacta; VT, ventriculus tecti mesencephalic; VTA, ventral tegmental area. Scale bars = 2.5 mm **(A,C)** and **(A′,C′)** and 1 mm **(B,D)** and **(B′,D′)**.

We examined the expression pattern of 5-*HTR1A* in sections A3.4 to A2.4 in P1 chick brainstems (Figure 2). We detected strong signals in the optic tectum (OT) in a layered manner (Figures 2A,C,A′,C′), nucleus intercolicularis (ICo) (Figures 2A,A′), oculomotor nucleus (OMN) (Figures 2A–D, Figures A′–D′), fasciculus longitudinalis medialis (FLM), and MR (Figures 2C,D,C′,D′), in consistency with our previous study (Fujita et al., 2022a). However, we sparsely detected cells with signals in the SNc, VTA, and A8 (Figures 2C,D,C′,D′).

### Expression of *5-HTR1B* in the chick brainstem

**FIGURE 3:**
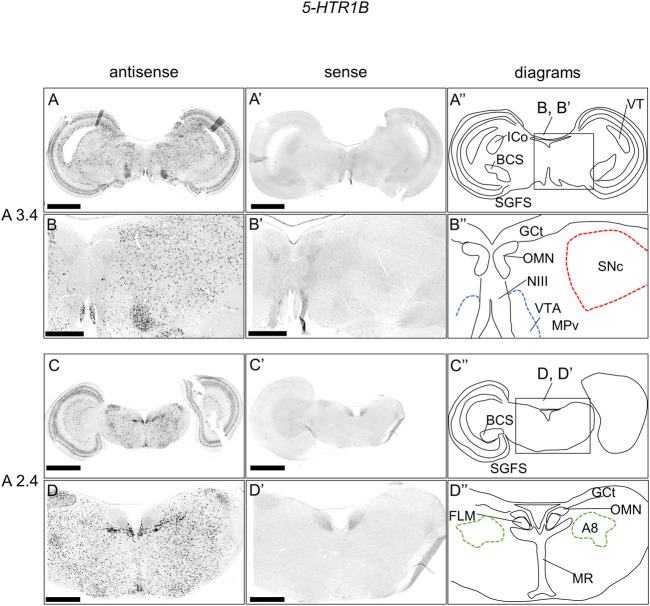
*In situ* hybridization of *5-HTR1B* in the P1 chick brainstem. Digoxigenin-labeled RNA antisense **(A–D)** and sense (**A′–D′**) *5-HTR1B* probes were used for *in situ* hybridization in coronal sections of P1 chick brainstems. To evaluate the expression patterns of *5-HTR1B*, we analyzed sections from five chick brainstems; representative images of chick brainstem sections are shown. **(B)** and **(B′)** and **(D)** and **(D′)** Magnified views of brain areas in the box in **(A′′)** and **(C′′)**. **(A′′–D′′)** Diagrams of coronal sections shown in the rightmost panels. The levels of expression in sections (A 3.4 and A 2.4) were in accordance with those mentioned in the Kuenzel and Masson’s chick atlas (Kuenzel and Masson, 1988). A8: A8 cell group; BCS, brachium colliculi superiors; FLM, fasciculus longitudinalis medialis; GCt, griseum centrale; ICo, nucleus intercolicularis; MR, median raphe; NIII, nervus oculomotorius; OMN, oculomotor nucleus; P1, post-hatch day 1; PPT, pedunculopontine tegmental nucleus; SGFS, stratum griseum et fibrosum superficiale; SNc, substantia nigra pars compacta; VT, ventriculus tecti mesencephalic; VTA, ventral tegmental area. Scale bars = 2.5 mm **(A,C)** and **(A′,C′)** and 1 mm **(B,D)** and **(B′,D′)**.

As with 5-*HTR1A*, we examined the expression pattern of 5-*HTR1B* in sections A3.4 to A2.4 in P1 chick brainstems (Figure 3). We detected strong signals in the OT in a layered manner and in a wide area of the brainstem, consistent with our previous findings (Figures 3A–D, Figures 3A′–D′) (Fujita et al., 2022a). In addition, we detected strong signals in the nucleus mesencephalicus proundus pars ventralis (MPv) (Figures 3A,B,A′,B′) and signals in the SNc, VTA, and A8 (Figures 3A–D, Figures 3A′–D′).

### Expression of *5-HTR1F* in the chick brainstem

**FIGURE 4:**
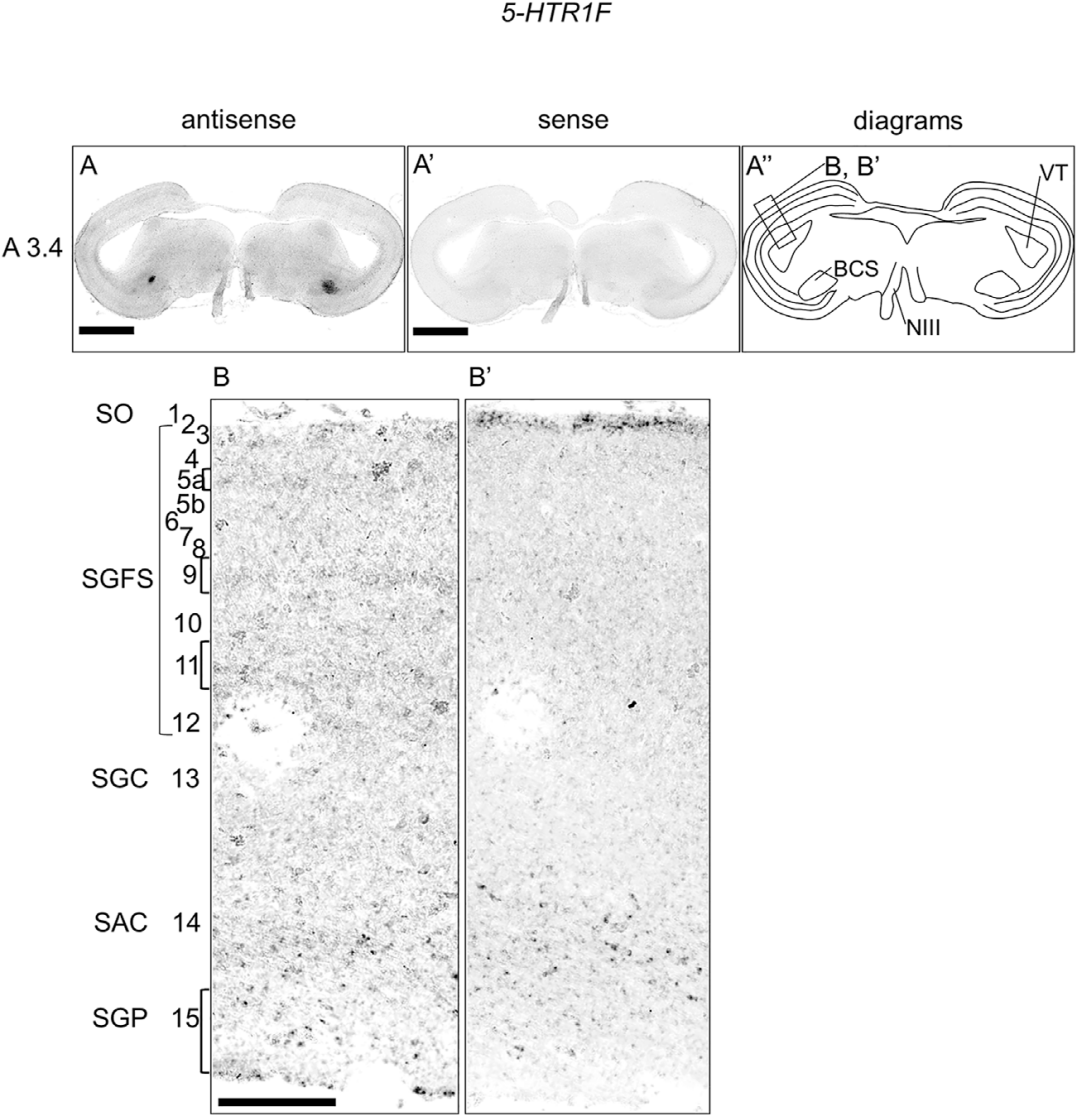
*In situ* hybridization of *5-HTR1F* in the P1 chick brainstem. Digoxigenin-labeled RNA antisense **(A,B)** and sense **(A′,B′)** *5-HTR1F* probes were used for *in situ* hybridization in coronal sections of P1 chick brainstems. To evaluate the expression patterns of *5-HTR1F*, we analyzed sections from three chick brainstems; representative images of chick brain sections are shown. **(A′′)** Diagrams of coronal sections shown in the rightmost panels. The levels of expression in sections (A3.4) were in accordance with those mentioned in the Kuenzel and Masson’s chick atlas (Kuenzel and Masson, 1988). **(B,B′)** Magnified views of the regions of optic tectum. Numbers represent tectal layers according to Ramon y Cajal (Ramon y Cajal, 1911). The leftmost alphabetical system of nomenclature was in accordance with that mentioned in the Kuenzel and Masson’s chick atlas (Kuenzel and Masson, 1988). BCS, brachium colliculi superiors; NIII, nervus oculomotorius; P1, post-hatch day 1; SAC, stratum album centrale; SGC, stratum griseum centrale; SGFS, stratum griseum et fibrosum superficiale; SGP, stratum griseum periventriculare; SO, stratum opticum. Scale bars = 2.5 mm **(A,A′)** and 250 µm **(B,B′)**.

We also examined the expression of 5-*HTR1F* in sections of P1 chick brainstems and detected signals in a part of the BCS and a weak layered pattern in the OT (Figures 4A,A′,A′′). When we observed the part of the OT in detail, we detected weak signals in the stratum griseum et fibrosum superficiale (SGFS; layers 5a, 9, and 11) and sparse signals in the stratum griseum periventriculare (SGP, layer 15) (Figures 4B,B′).

### Expression of *5-HTR2A* and *5-HTR2C* in the chick brainstem

**FIGURE 5:**
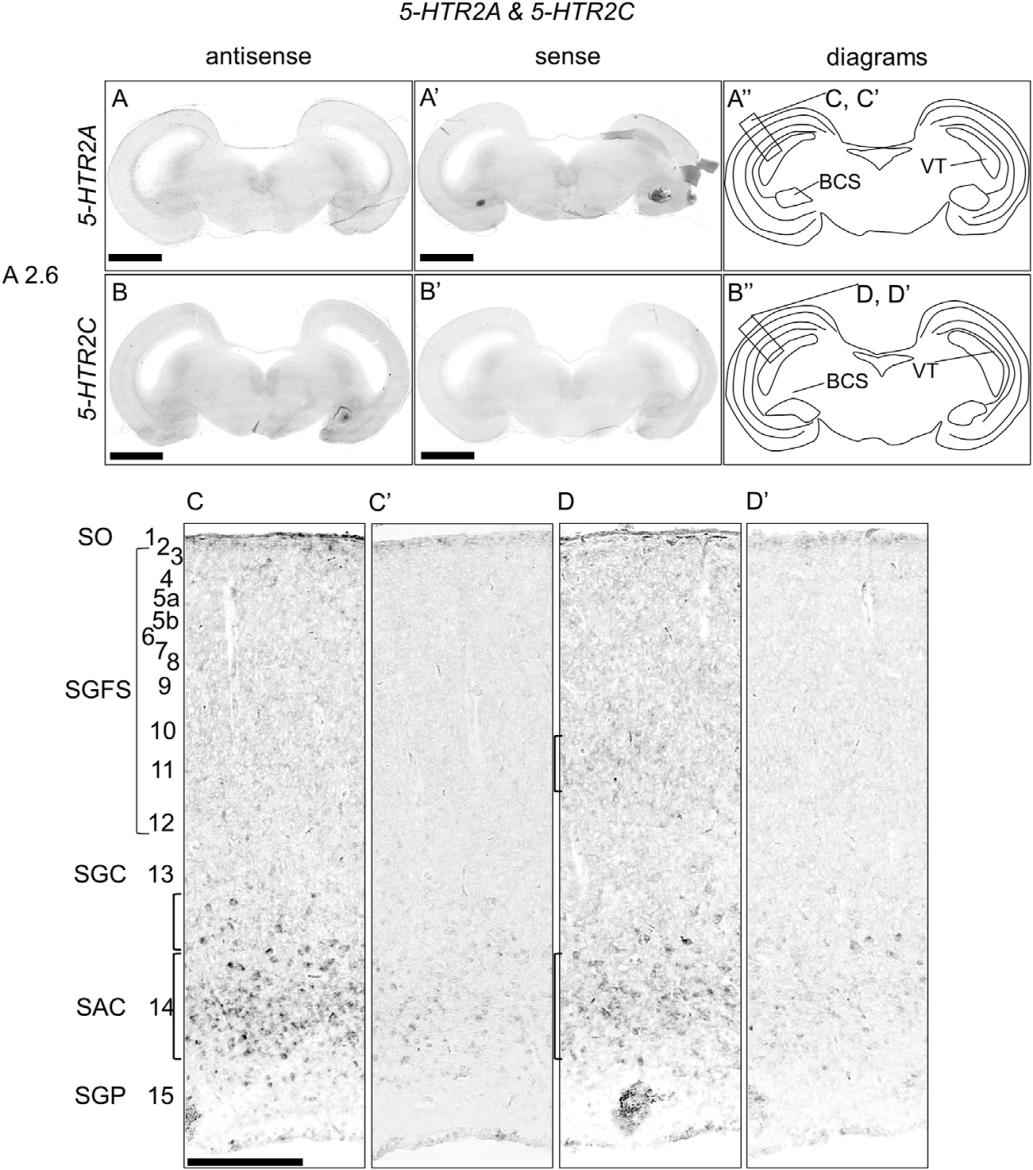
*In situ* hybridization of *5-HTR2A* and *5-HTR2C* in the P1 chick brainstem. Digoxigenin-labeled RNA antisense [*5-HTR2A*, **(A,C)**, and *5-HTR2C,* **(B,D)**] and sense [*5-HTR2A*, **(A′,C′)**, and *5-HTR2C,* **(B′,D′)**] probes were used for *in situ* hybridization in coronal sections of P1 chick brainstems. To evaluate the expression patterns of *5-HTR2A* and *5-HTR2C*, we analyzed sections from four chick brainstems for *5-HTR2A* and 4 for *5-HTR2C*; representative images of chick brainstem sections are shown. **(A′′,B′′)** Diagrams of coronal sections shown in the rightmost panels. The levels of expression in sections (A2.6) were in accordance with those mentioned in the Kuenzel and Masson’s chick atlas (Kuenzel and Masson, 1988). **(C,D)** and **(C′,D′)** Magnified views of the regions of optic tectum. Numbers represent tectal layers according to Ramon y Cajal (Ramon y Cajal, 1911). The leftmost alphabetical system of nomenclature was in accordance with that mentioned in the Kuenzel and Masson’s chick atlas (Kuenzel and Masson, 1988). BCS, brachium colliculi superiors; NIII, nervus oculomotorius; P1, post-hatch day 1; SAC, stratum album centrale; SGC, stratum griseum centrale; SGFS, stratum griseum et fibrosum superficiale; SGP, stratum griseum periventriculare; SO, stratum opticum. Scale bars = 2.5 mm **(A,B)** and **(A′,B′)** and 250 µm **(C,D)** and **(C′,D′)**.

We examined the expression of *5-HTR2A* and *5-HTR2C* in sections of P1 chick brainstems but did not detect any clear expression patterns (Figures 5A,B,A′,B′,A′′,B′′). However, when we observed the part of the OT in detail, we detected *5-HTR2A* signals in the stratum album centrale (layer 14) and a part of SGC (layer 13) (Figures 5C,C′). In addition, we detected *5-HTR2C* signals in the SGFS (layer11) and SAC (layer 14) (Figure 5D,D′).

### Expression of *5-HTR1D, 5-HTR1E, 5-HTR2B, 5-HTR3A, 5-HTR4, 5-HTR5A,* and *5-HTR7* in the chick brainstem

We examined the expression of *5-HTR1D, 5-HTR1E, 5-HTR2B, 5-HTR3A, 5-HTR4, 5-HTR5A,* and *5-HTR7* in sections around A3.4 to A2.4 in P1 chick brainstems but did not detect any signals, suggesting that either the levels of expression of *5-HTR* genes were very low or cells expressing *5-HTR* genes were very rare. This was supported by the results of RT-PCR using cDNA synthesized from the chick brainstems (Supplementary Figure S3).

### Double ISH analysis of *5-HTR1A* and *5-HTR1B* with dopaminergic neuron marker genes in the chick brainstem

**FIGURE 6:**
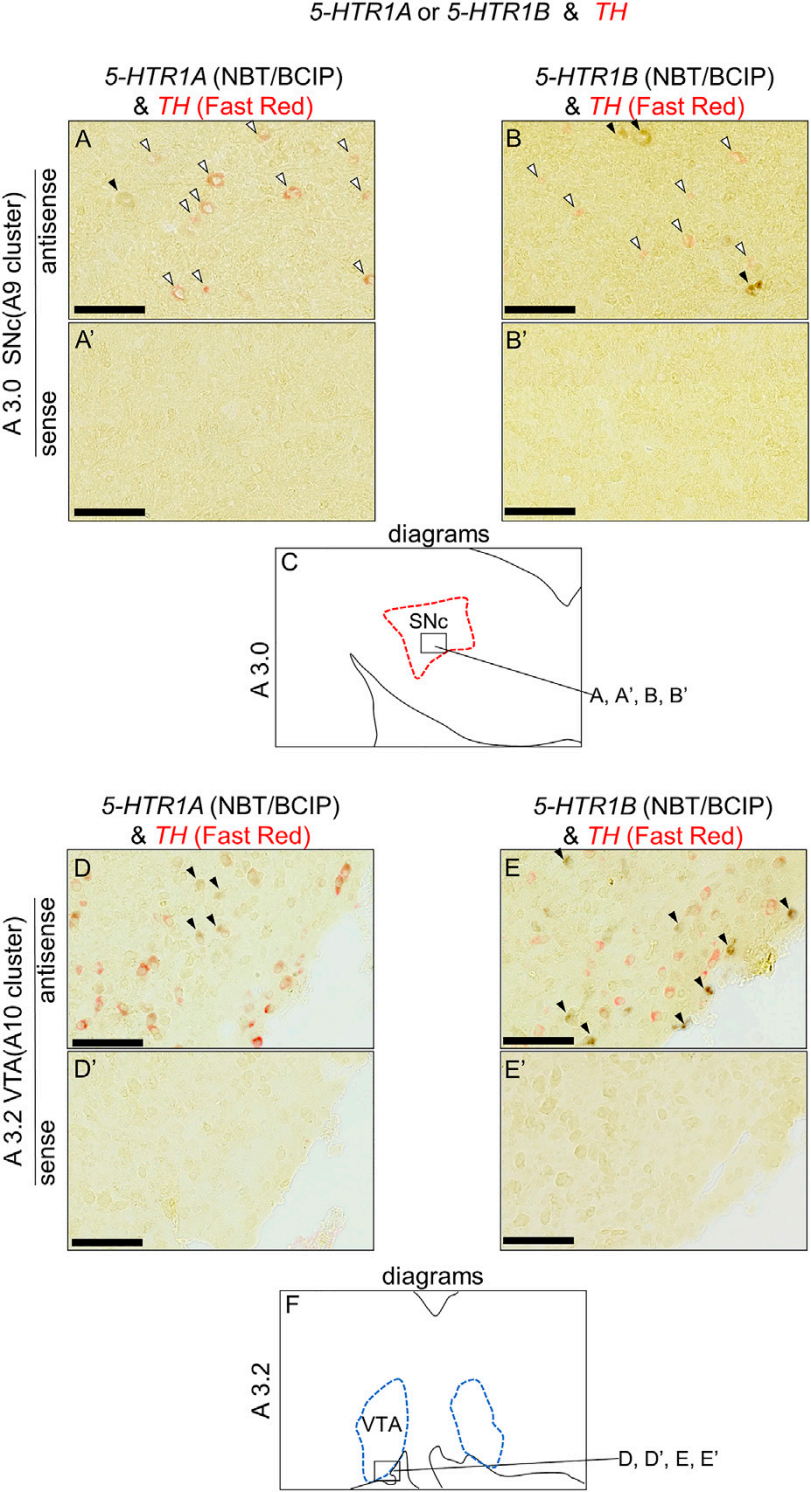
Double *in situ* hybridization of *5-HTR1A* or *5-HTR1B* and *TH* in the SNc and VTA of the P1 chick brainstem. Digoxigenin-labeled (*5-HTR1A* and *5-HTR1B*) and fluorescein-labeled *TH* RNA antisense **(A,B,D,E)** and sense **(A′,B′,D′,E′)** probes were used for double *in situ* hybridization in coronal sections of P1 chick brainstems. **(A,B)** and **(A′,B′)** Magnified views of dopaminergic nuclei in the box in **(C)**. Black arrowheads indicate *5-HTR1A* or *5-HTR1B* signals, whereas white arrowheads indicate *TH* signals. **(C)** Diagrams of coronal sections shown in the A3.0 sections (Supplementary Figure S4). **(D,E)** and **(D′,E′)** Magnified views of dopaminergic nuclei in the box in **(F)**. Black arrowheads indicate *5-HTR1A* or *5-HTR1B* signals, whereas white arrowheads indicate *TH* signals. **(F)** Diagrams of coronal sections shown in the A3.2 sections (Supplementary Figure S4). SNc, substantia nigra pars compacta; VTA, ventral tegmental area. Scale bars = 100 µm.

We found that *5-HTR1A* and *5-HTR1B* were expressed in the A8, SNc, and VTA. Subsequently, we examined whether *5-HTR*-expressing cells were dopaminergic neurons or not. We performed a double ISH analysis in sections A3.0 and A3.2, including SNc and VTA, to test whether *5-HTR1A-* or *5-HTR1B*-expressing cells expressed a dopaminergic neuron marker gene, *TH* (Figure 6). We detected cells with alternating *5-HTR1A* or *TH* signals, but did not detect any double positive cells for *5-HTR1A* and *TH* in the SNc (Figures 6A,A′) and the VTA (Figures 6D,D′). Similarly, we detected cells with alternating *5-HTR1B* or *TH* signals but no double positive cells for *5-HTR1B* and *TH* in the SNc (Figures 6B,B′) and the VTA (Figures 6E,E′).

## Discussion

### Distribution and molecular property of dopaminergic neurons in the midbrain of chicks

In the present study, we described the distribution of midbrain dopaminergic neurons in the brainstem of chicks, which were characterized by the expression of the chick orthologs of mammalian dopaminergic neuron-related genes, *TH* and *DDC*, and the lack of expression of the chick ortholog of mammalian noradrenergic neuron-related gene, *DBH* (Figure 1). We clearly showed that midbrain dopaminergic neurons were distributed in the A8, A9 (SNc), and A10 (VTA), in agreement with previous studies (Ikeda and Goto, 1971; Dube and Parent, 1981; Guglielmone and Panzica, 1984; Moons et al., 1994). Generally, ISH analysis detects only the cell bodies of neurons, making it suitable for the purpose of mapping the distributions of dopaminergic neuron cells and evaluating heterogeneity.

In addition, we showed that dopaminergic neurons existed in the DR (Figures 1P–S, Figures 1P′–S′). In mammals, the DR dopaminergic neurons exist as a specialized midbrain dopaminergic subsystem that encodes incentive salience and controls the expression of incentive memory, with focal projections mainly to the central amygdala and the bed nucleus of stria terminalis (Hasue and Shammah-Lagnado, 2002; Poulin et al., 2014; Lin et al., 2020; Lin et al., 2021). To our knowledge, not much is known about DR dopaminergic neurons in birds. In the future, through the use of neuronal tracers, it will be interesting to determine whether the functions and projection targets of DR dopaminergic neurons are conserved between birds and mammals.

In mammals, the numbers and distribution of dopaminergic neurons in the midbrain dopaminergic nuclei have been extensively quantified (Swanson, 1982; German and Manaye, 1993; Nair-Roberts et al., 2008). For example, Swanson (1982) estimated that two-thirds of cells were dopaminergic in the rat VTA. Furthermore, considerable heterogeneities have been detected within the nuclei, which contain glutamatergic and GABAergic neurons in addition to dopaminergic neurons (Nair-Roberts et al., 2008; Lammel et al., 2014). However, currently, there is no data on what type of cells exist in the dopaminergic nuclei of birds and in what proportions. Yet, our data indicated that a *TH*-positive/*DDC*-positive/*DBH*-negative cell population exists in many of these dopaminergic nuclei (VTA, SNc, and A8). Our results suggested that the proportion of dopaminergic neurons in the dopaminergic nuclei of birds is not inconsistent with those of mammals. However, a more detailed estimation of avian dopaminergic nuclei will be necessary in the future.

In a previous study, Lovell et al. (2015) showed that the *DAT* gene was deleted in the genomes of sauropsids (birds and reptiles) and that *NAT* was expressed in the SNc and VTA of songbirds, suggesting that NAT compensates for the function of DAT in sauropsids (Lovell et al., 2015). We found that all nuclei containing dopaminergic neurons in the chick midbrain also expressed *NAT*, suggesting that the expression pattern of *NAT* was a general molecular property of the midbrain dopaminergic neurons in the chick. Hence, our results supported the theory of NAT compensating the function of DAT.

### Serotonergic regulation *via* 5-HTR1A and 5-HTR1B in chick midbrain dopaminergic nuclei

**FIGURE 7:**
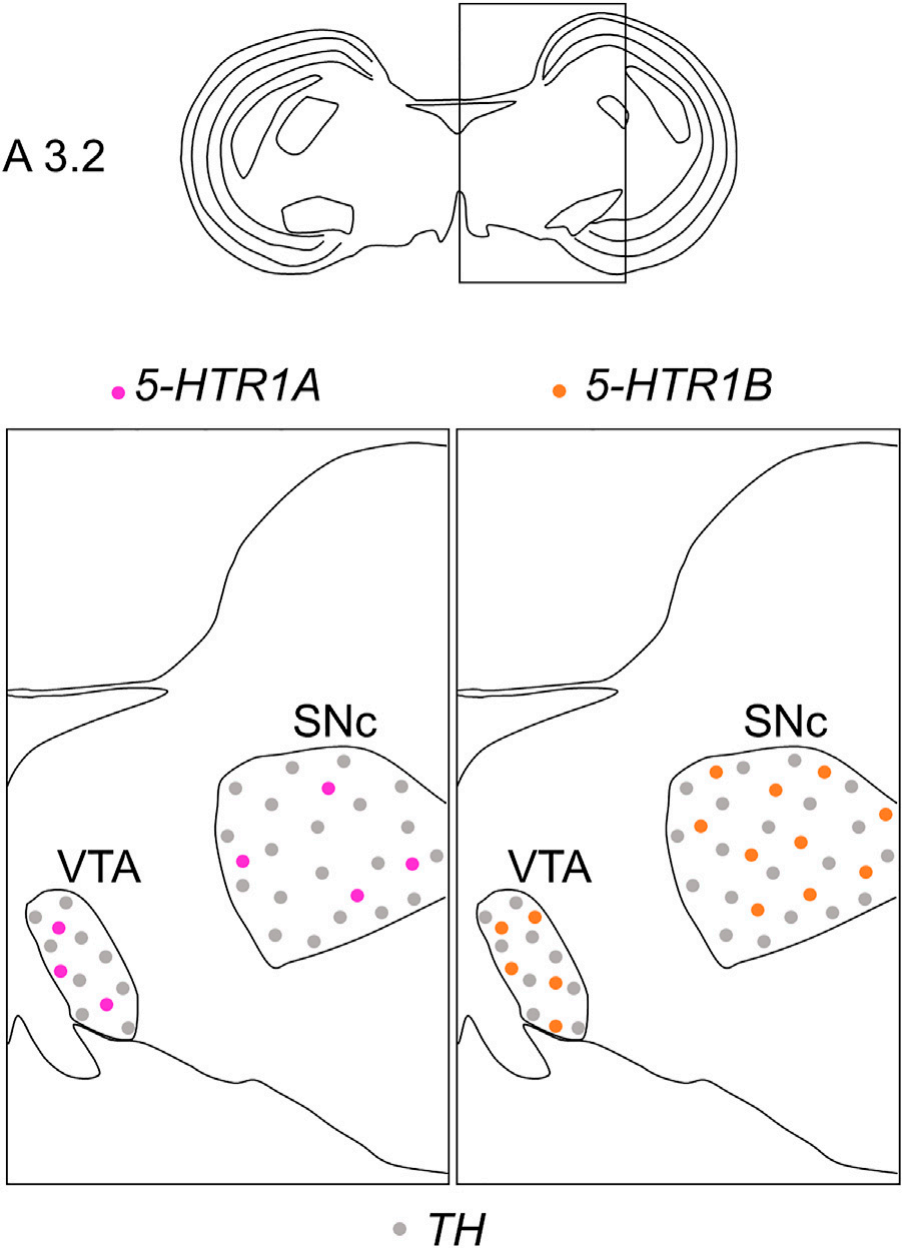
Schematic summary of the expression patterns of *5-HTR1A* or *5-HTR1B* and *TH* in dopaminergic nuclei in the P1 chick midbrain. Representative expression patterns in sections around A 3.2 are exhibited in colored areas (magenta, *5-HTR1A*; orange, *5-HTR1B*; grey, *TH*). The alternating dotted pattern indicates that the cells were sparsely distributed with alternating *5-HTR1A* (or *5-HTR1B*) and *TH* signals but no double positive signal. The levels of expression in sections were in accordance with those mentioned in the Kuenzel and Masson’s chick atlas (Kuenzel and Masson, 1988). P1, post-hatch day 1; SNc, substantia nigra pars compacta; VTA, ventral tegmental area.

We found that *5-HTR1A* was expressed in the A8, SNc, and VTA in the chick midbrain (Figure 2), suggesting that chick dopaminergic nuclei are modulated by serotonin through 5-HTR1A. We also found that *5-HTR1A*-expressing cells in the dopaminergic nuclei did not overlap with *TH*-expressing neurons in the chick midbrain (Figure 7). In mammals, *5-Htr1a* was not strongly detected in dopaminergic nuclei (Pompeiano et al., 1992; Burnet et al., 1995; Pasqualetti et al., 1996; Vilaró et al., 2020). In addition, using neuronal cell type markers revealed that *5-Htr1a*-expressing cells did not overlap with *Th*-expressing neurons but with glutamatergic or GABAergic neurons in the mouse rostral brainstem (Bonnavion et al., 2010). Accordingly, *5-HTR1A*-expressing cells in the chick dopaminergic nuclei might be glutamatergic or GABAergic neurons. Our findings regarding the expression of *5-HTR1A* in dopaminergic nuclei will facilitate the elucidation of the mechanism by which 5-HTR1A-mediated regulation of dopaminergic nuclei affects behavior in chicks and the understanding of the interactions between dopaminergic and serotonergic systems in birds.

We found that *5-HTR1B* was clearly expressed in the A8, SNc, and VTA in the chick midbrain (Figure 3), further suggesting that chick dopaminergic nuclei are modulated by serotonin through 5-HTR1B. We also found that *5-HTR1B*-expressing cells in the dopaminergic nuclei did not overlap with *TH*-expressing neurons in the chick midbrain (Figure 7), suggesting that *5-HTR1B*-expressing cells in the dopaminergic nuclei were glutamatergic or GABAergic neurons. It is possible that *5-HTR1B*-expressing glutamatergic- or GABAergic neurons, which constitute a local microcircuit in dopaminergic nuclei, might regulate dopaminergic neurons in the chick midbrain. In mammals, high density signals of *5-HTR1B* were detected in the broad brain areas, including the substantia nigra and VTA using radioligands (Bonaventure et al., 1997; Varnas et al., 2001). On the contrary, ISH analysis revealed the lack of signals of *5-HTR1B* specific RNA probes in the substantia nigra and VTA, suggesting that the high densities of 5-Htr1b protein found in these regions of mismatch were located on axon terminals of extrinsic origin, the presynaptic receptors, or presynaptic autoreceptors of serotonergic innervations (Bruinvels et al., 1994; Varnas et al., 2005). These findings suggested that dopaminergic neurons in the midbrain dopaminergic nuclei are regulated by serotonin *via 5-HTR1B*, and these properties might be evolutionarily conserved between birds and mammals. Differences in the expression of *5-HTR1B* between bird and mammalian dopaminergic nuclei have suggested that the serotonin-regulated local microcircuitries in the dopaminergic nuclei between bird and mammals are different.

In this study, other *5-HTR* subfamily genes except *5-HTR1A* and *5-HTR1B* were not detected in the dopaminergic nuclei in chick midbrains. These results suggested that the levels of expression of these 5-HTR genes were very low or cells expressing 5-HTR genes were very rare. The serotonergic regulation to the reward processing *via* 5-HTR2A, 5-HTR2C, and 5-HTR3A in mammals has attracted increased attention (Liu et al., 2020; Peters et al., 2021). Moreover, using a combination of immunolabeling and ISH with radioisotope-labeled probe autoradiography revealed that *5-HTR3A* was expressed on Th-positive neurons in the VTA in mice (Wang et al., 2019). Generally, autoradiography using radioisotope-labeled probe can achieve higher sensitivity than the conventional chemical coloring ISH method. In addition, recent single-cell RNA sequencing studies of dopaminergic nuclei showed that the levels of expression of *5-Htr* genes were very low (Saunders et al., 2018; Huang et al., 2019; Tiklova et al., 2019; Poulin et al., 2020). This was consistent with our findings that the levels of expression of *5-Htr* genes were too low to detect by our ISH system in this study. In the future, using more sensitive detection systems will clarify whether 5-HTR2A, 5-HTR2C, and 5-HTR3A are expressed and regulated in the avian dopaminergic system.

### Expression patterns of *5-HTR1F, 5-HTR2A* and *5-HTR2C* in the OT layers

The optic tectum (OT) of birds has been extensively developed, exhibiting a high degree of lamination. The OT is a major component of the tectofugal pathway, which includes the retina, OT, nucleus rotundus, and entopallium, which is thought to be the most important visual pathway in birds (Wylie et al., 2009). The OT has 15 perceptible layers and a strict separation between visual input and output. In general, inputs from the retina terminate in superficial layers, whereas the main output from the OT to the nucleus rotundus comes from projection neurons in deeper layers, especially layer 13 (SGC) (Ramon y Cajal, 1911; Luksch, 2003). In a previous study, we showed that the expression patterns of *5-HTR1A* and *5-HTR1B* in the OT layers were mutually exclusive (Fujita et al., 2022a). In this study, to deepen our understanding of serotonergic regulation in the OT, we carefully and comprehensively performed ISH analysis and evaluated the expression of *5-HTR* subtypes in the chick OT. We found that *5-HTR1F*-expressing cells were distributed in layers 5a, 9, 11, and 15 (Figures 4B,B′), *5-HTR2A*-expressing cells were distributed in layer 14 and a part of layer 13 (Figures 5C,C′), and *5-HTR2C*-expressing cells were distributed in layers 11 and 14 (Figures 5D,D′). In addition to *5-HTR1A* and *5-HTR1B* (Fujita et al., 2022a), *5-HTR1F*, *5-HTR2A*, and *5-HTR2C* might play important roles in the modulation of the function of neurons, including visual processing in the tectofugal pathway. For example, we previously showed that *5-HTR1F* was preferentially expressed in the interstitial part of the hyperpallium (IHA) in the chick telencephalon (Fujita et al., 2022b). The IHA is part of the homologous region to the mammalian neocortex (Fernandez et al., 1998; Puelles et al., 2000) with projection terminals of sensory information from the thalamus, and is thus considered equivalent to layer IV of the mammalian neocortex (Bagnoli and Burkhalter, 1983; Miceli and Repérant, 1985; Miceli et al., 1990; Atoji et al., 2018; Atoji and Wild, 2019). Here, we found that *5-HTR1F* was expressed in layers 5a and 9 of the chick OT (Figures 4B,B′). Recently, a detailed morphological and physiological study of neurons in layer 9 of chicken OT demonstrated the nature of input received in retinal projection *via* glutamatergic synapses (Kloos et al., 2019). Taken together, it might be possible that *5-HTR1F*-expressing neurons in the IHA and layers 5a and 9 in the OT share a common function in processing sensory input under serotonergic modulation. In addition, a previous study that used immunohistochemistry reported that 5-HTR2A was intensely expressed throughout layer 13 in the chicken OT (Metzger et al., 2006). Our data clearly showed that *5-HTR2A*-expressing cells were distributed in a part of the deep side of layer 13 and throughout layer 14 (Figures 5C,C′), in consistency with a previous study regarding the output deep layer (Metzger et al., 2006). This discordance between our data and those of the previous study is not clear, but the comparison of the layer of expression of *5-HTR2A* with that of *5-HTR1A*, which has been shown to be more strongly and sharply expressed in layer 13, could clarify the exact expression layer.

## Conclusion

We have comprehensively described the distribution of dopaminergic neurons in the A8, SNc, and VTA of the chick midbrain and found that *5-HTR1A* and *5-HTR1B* were expressed in dopaminergic nuclei. 5-HTR1A and 5-HTR1B, which are mainly associated with the dopaminergic system, might be involved in the regulation of motor control and motivation-related behavior. In addition, we found that *5-HTR1F*, *5-HTR2A*, and *5-HTR2C* were expressed in the OT in layer-selective manners. Our findings can facilitate the improvement of our understanding of the interactions between dopaminergic and serotonergic systems in birds and the serotonergic regulation of OT neural circuits.

## Data Availability

The original contributions presented in the study are included in the article/Supplementary Material, further inquiries can be directed to the corresponding author.
