# Supplementary material for: Dopaminergic nuclei in the chick midbrain express serotonin receptor subfamily genes
Source: Front Physiol. 2022 Nov 8;13:1030621. doi: 10.3389/fphys.2022.1030621 (PMC9679639; doi:10.3389/fphys.2022.1030621)

Supplemental Table 1. Subject summary used in this study

	chick #s											
	#1	#2	#3	#4	#5	#6	#7	#8	#9	#10	#11	#12
probes												
TH	○	○	○	○	○	○	○	○	○	○	-	-
DDC	○	○	○	○	○	○	○	○	-	-	-	-
NAT	○	○	○	○	○	○	○	-	-	-	-	-
DBH	○	○	○	○	○	○	-	-	○	-	-	-
5-HTR1A	○	○	○	-	○	-	-	-	○	-	-	-
5-HTR1B	○	○	○	-	○	-	-	-	○	-	-	-
5-HTR1D	○	○	○	-	-	-	-	-	-	-	-	-
5-HTR1E	○	○	○	-	-	-	-	-	-	-	-	-
5-HTR1F	○	-	○	○	-	-	-	-	-	-	-	-
5-HTR2A	○	○	○	○	-	-	-	-	-	-	-	-
5-HTR2B	-	○	-	○	-	-	-	-	○	-	-	-
5-HTR2C	○	○	○	○	-	-	-	-	-	-	-	-
5-HTR3A	○	-	○	-	○	-	-	-	○	-	-	-
5-HTR4	○	-	-	○	-	-	-	-	○	-	-	-
5-HTR5A	-	○	-	○	-	-	-	-	○	-	-	-
5-HTR7	○	-	-	○	-	-	-	-	○	-	-	-
RT-PCR	-	-	-	-	-	-	-	-	-	-	○	○

Supplemental Table 2. Overview of *5-HTR* subfamily RNA probes used in this study

**Accession number**	**Gene symbol**	**Molecular characteristics**	**Probe preparation**
NM_001170528.1	*5-HTR1A*	GPCR	Fujita, et al. (2020)
XM_015284634.2	*5-HTR1B*	GPCR	Fujita, et al. (2020)
XM_015297583.1	*5-HTR1D*	GPCR	Fujita, et al. (2022a)
XM_015284709.2	*5-HTR1E*	GPCR	Fujita, et al. (2022a)
XM_004938334.3	*5-HTR1F*	GPCR	Fujita, et al. (2022a)
XM_025151250.1	*5-HTR2A*	GPCR	Fujita, et al. (2020)
XM_025153310.1	*5-HTR2B*	GPCR	Fujita, et al. (2022a)
XM_004940651.3	*5-HTR2C*	GPCR	Fujita, et al. (2020)
XM_004948063.3	*5-HTR3A*	Ligand-gated ion channel	Fujita, et al. (2020)
XM_015293658.2	*5-HTR4*	GPCR	Fujita, et al. (2020)
XM_425970.3	*5-HTR5A*	GPCR	Fujita, et al. (2022a)
XM_015288394.2	*5-HTR7*	GPCR	Fujita, et al. (2022a)

5-HTR: 5-hydroxytriptamine receptor; GPCR: G protein-coupled receptor

Supplemental Figure 1. *5-HTR* probes sequencing and alignment results

5-HTR1A

```
>Gallus gallus 5-hydroxytryptamine receptor 1A (HTR1A), mRNA
Sequence ID: NM_001170528.1 Length: 1335
>Gallus gallus 5-hydroxytryptamine receptor 1A precursor (HTR1A) mRNA, complete cds
Sequence ID: GU189388.1 Length: 1335
Range 1: 79 to 815

Score:1362 bits(737), Expect:0.0,
Identities:737/737(100%),  Gaps:0/737(0%), Strand: Plus/Plus

Query  1    AACACTACCTCCCCAGAACGCTCCCCCGAGGGGGAAGGTGGCCCCGGTCTCGCCGAGGTG  60
            ||||||||||||||||||||||||||||||||||||||||||||||||||||||||||||
Sbjct  79   AACACTACCTCCCCAGAACGCTCCCCCGAGGGGGAAGGTGGCCCCGGTCTCGCCGAGGTG  138

Query  61   ACCCTGGGCTACCAGCTGCTCACCTCCCTGCTCCTGGGCACGCTCATCCTGTGCGCCGTG  120
            ||||||||||||||||||||||||||||||||||||||||||||||||||||||||||||
Sbjct  139  ACCCTGGGCTACCAGCTGCTCACCTCCCTGCTCCTGGGCACGCTCATCCTGTGCGCCGTG  198

Query  121  AGCGGCAACGCCTGCGTGATCGCTGCCATCGCCCTGGAGCGCTCCCTGCAAACCGTGGCC  180
            ||||||||||||||||||||||||||||||||||||||||||||||||||||||||||||
Sbjct  199  AGCGGCAACGCCTGCGTGATCGCTGCCATCGCCCTGGAGCGCTCCCTGCAAACCGTGGCC  258

Query  181  AACTATCTCATCGGCTCGCTGGCCGTCACCGACCTCATGGTGTCCGTGCTGGTGCTGCCC  240
            ||||||||||||||||||||||||||||||||||||||||||||||||||||||||||||
Sbjct  259  AACTATCTCATCGGCTCGCTGGCCGTCACCGACCTCATGGTGTCCGTGCTGGTGCTGCCC  318

Query  241  ATGGCGGCCCTCTACCAGGTGCTGAACAAGTGGACTCTGGGGCAGGTCATCTGCGATATC  300
            ||||||||||||||||||||||||||||||||||||||||||||||||||||||||||||
Sbjct  319  ATGGCGGCCCTCTACCAGGTGCTGAACAAGTGGACTCTGGGGCAGGTCATCTGCGATATC  378

Query  301  TTCATCTCGCTGGACGTGCTGTGCTGCACCTCTTCCATCCTGCACCTGTGCGCCATCGCC  360
            ||||||||||||||||||||||||||||||||||||||||||||||||||||||||||||
Sbjct  379  TTCATCTCGCTGGACGTGCTGTGCTGCACCTCTTCCATCCTGCACCTGTGCGCCATCGCC  438

Query  361  TTGGACAGGTACTGGGCCATCACGGACCCCATCGACTATGTCAACAAGCGAACTCCCCGC  420
            ||||||||||||||||||||||||||||||||||||||||||||||||||||||||||||
Sbjct  439  TTGGACAGGTACTGGGCCATCACGGACCCCATCGACTATGTCAACAAGCGAACTCCCCGC  498

Query  421  CGGGCCGCCGTGCTCATCAGCCTGACCTGGCTCATCGGCTTCTTGATATCCATCCCGCCC  480
            ||||||||||||||||||||||||||||||||||||||||||||||||||||||||||||
Sbjct  499  CGGGCCGCCGTGCTCATCAGCCTGACCTGGCTCATCGGCTTCTTGATATCCATCCCGCCC  558

Query  481  ATGCTGGGCTGGAGGACACCCGAGGACCGCTCAAATCCTGACGCCTGCACCATCAGCAAG  540
            ||||||||||||||||||||||||||||||||||||||||||||||||||||||||||||
Sbjct  559  ATGCTGGGCTGGAGGACACCCGAGGACCGCTCAAATCCTGACGCCTGCACCATCAGCAAG  618

Query  541  GACCACGGGTACACCATCTACTCCACCTTCGGCGCCTTCTACATCCCGCTGCTCCTCATG  600
            ||||||||||||||||||||||||||||||||||||||||||||||||||||||||||||
Sbjct  619  GACCACGGGTACACCATCTACTCCACCTTCGGCGCCTTCTACATCCCGCTGCTCCTCATG  678

Query  601  CTGGTGCTCTACGGCCGCATCTTCAAAGCAGCCCGCTTCAGGATCCGCAAGACAGTCAAG  660
            ||||||||||||||||||||||||||||||||||||||||||||||||||||||||||||
Sbjct  679  CTGGTGCTCTACGGCCGCATCTTCAAAGCAGCCCGCTTCAGGATCCGCAAGACAGTCAAG  738

Query  661  AAAGCAGAAAAGAAGAAAATCGCCGACACTTGCCTCACCCTCTCCCCGGCCGCCCTGCAG  720
            ||||||||||||||||||||||||||||||||||||||||||||||||||||||||||||
Sbjct  739  AAAGCAGAAAAGAAGAAAATCGCCGACACTTGCCTCACCCTCTCCCCGGCCGCCCTGCAG  798

Query  721  AAGAAAAGCAACGGGGA  737
            |||||||||||||||||
Sbjct  799  AAGAAAAGCAACGGGGA  815
```

5-HTR1B

```
>PREDICTED: Gallus gallus 5-hydroxytryptamine receptor 1B (HTR1B), transcript variant X1, mRNA
Sequence ID: XM_046914126.1 Length: 2437
Range 1: 1104 to 1811

Score:1288 bits(697), Expect:0.0,
Identities:707/711(99%),  Gaps:3/711(0%), Strand: Plus/Plus

Query  1     TCACGTGGCTGGGATATCTCAACTCCCTCATCAACCCCATCATCTATACCATGTCTAACG  60
             ||||||||||||||||||||||||||||||||||||||||||||||||||||||||||||
Sbjct  1104  TCACGTGGCTGGGATATCTCAACTCCCTCATCAACCCCATCATCTATACCATGTCTAACG  1163

Query  61    AAGACTTCAAGCAAGCTTTCCACAAACTCATCCGTTTCCGCTGCACAGGCTGAACATTTG  120
             ||||||||||||||||||||||||||||||||||||||||||||||||||||||||||||
Sbjct  1164  AAGACTTCAAGCAAGCTTTCCACAAACTCATCCGTTTCCGCTGCACAGGCTGAACATTTG  1223

Query  121   GAATGCGATGTGTTCCCCGGGGCCGCCCCCGCGCACGCCCGTGCCCGATGCCACAGAATC  180
             ||||||||||||||| ||||||||||||||||||||||||||||||||||||||||||||
Sbjct  1224  GAATGCGATGTGTTCTCCGGGGCCGCCCCCGCGCACGCCCGTGCCCGATGCCACAGAATC  1283

Query  181   AAGTTGCTATTGTAAAGACCCACTGCTTGAAACTTCCCCAAGCCCAGTTGCCAGACTCAT  240
             ||||||||||||||||||||||||||||||||||||||||||||||||||||||||||||
Sbjct  1284  AAGTTGCTATTGTAAAGACCCACTGCTTGAAACTTCCCCAAGCCCAGTTGCCAGACTCAT  1343

Query  241   GATGCTGCAAAAAAACGTCAATACATACTGCCAGCAAACTGCTCAGCTCTGCATTTCAGTG  300
             ||||||||||||||||||||||||||||||||||||||||||||||||||||||||||||
Sbjct  1344  GATGCTGCAAAAAAACGTCAATACATACTGCCAGCAAACTGCTCAGCTCTGCATTTCAGTG  1403

Query  301   TAATGACTCTGGCAGAGATGCTGTTCCAGTAGTCACGCAGATCAGTTGCaaaaaaaGAGG  360
             ||||||||||||||||||||||||||||||||||||||||||||||||||||||||||||
Sbjct  1404  TAATGACTCTGGCAGAGATGCTGTTCCAGTAGTCACGCAGATCAGTTGCAAAAAAAGAGG  1463

Query  361   GCAGCCTCGAATCAAGGTCTATAATTCATCCGGACTGAAGAAAAAGCTTGAGAGTAAACT  420
             ||||||||||||||||||||||||||||||||||||||||||||||||||||||||||||
Sbjct  1464  GCAGCCTCGAATCAAGGTCTATAATTCATCCGGACTGAAGAAAAAGCTTGAGAGTAAACT  1523

Query  421   CAATTTATTTTGCAGGCTTAACTTCTTAATTTGTTCTCCGGCCAGTTGTAGGGGTGTGCA  480
             ||||||||||||||||||||||||||||||||||||||||||||||||||||||||||||
Sbjct  1524  CAATTTATTTTGCAGGCTTAACTTCTTAATTTGTTCTCCGGCCAGTTGTAGGGGTGTGCA  1583

Query  481   CAAGAGCACAGTTGTCTCTGTCTGTGTTGGTAGCGTAGTTGTACTTCTGCACACCTGTAA  540
             ||||||||||||||||||||||||||||||||||||||||||||||||||||||||||||
Sbjct  1584  CAAGAGCACAGTTGTCTCTGTCTGTGTTGGTAGCGTAGTTGTACTTCTGCACACCTGTAA  1643

Query  541   TTTCTTTCTTTCAGTGGAAAGAGTTGCTCACTCCTTATGAGCTCTAGAAGGGGaaaaaaa  600
             |||||||||||||||||||||||||||||||||||||||||||||||||||||   ||||
Sbjct  1644  TTTCTTTCTTTCAGTGGAAAGAGTTGCTCACTCCTTATGAGCTCTAGAAGGGG---AAAA  1700

Query  601   aaaaacaaaaccacaacgcaacaaaaacagctttaaaaaagaaaaaGGGGAGCATCACAG  660
             ||||||||||||||||||||||||||||||||||||||||||||||||||||||||||||
Sbjct  1701  AAAAACAAAACCACAACGCAACAAAAACAGCTTTAAAAAAGAAAAAGGGGAGCATCACAG  1760

Query  661   AAAACTCTGTGCACGACTCCTTCAGCTGAATGTGTGTGAGAAGTGGCAGTG  711
             |||||||||||||||||||||||||||||||||||||||||||||||||||
Sbjct  1761  AAAACTCTGTGCACGACTCCTTCAGCTGAATGTGTGTGAGAAGTGGCAGTG  1811
```

5-HTR1D

```
>PREDICTED: Gallus gallus 5-hydroxytryptamine (serotonin) receptor 1D, G protein-coupled (HTR1D), mRNA
Sequence ID: XM_040690105.2 Length: 3213
Range 1: 904 to 1607

Score:1290 bits(698), Expect:0.0,
Identities:702/704(99%),  Gaps:0/704(0%), Strand: Plus/Minus

Query  1     TTCGTGAAGGCTTGCGTTAATGGAGCAGAGGGAAGACCCAGCAGAGCCAGTTATCAGGTG  60
             ||||||||||||||||||||||||||||||||||||||||||||||||||||||||||||
Sbjct  1607  TTCGTGAAGGCTTGCGTTAATGGAGCAGAGGGAAGACCCAGCAGAGCCAGTTATCAGGTG  1548

Query  61    TGCAGTAGTGAATCGTTTCCCATACAGTGAGGGTGGCTTCAGGATCCTGGATCGAGCTGC  120
             ||||||||||||||||||||||||||||||||||||||||||||||||||||||||||||
Sbjct  1547  TGCAGTAGTGAATCGTTTCCCATACAGTGAGGGTGGCTTCAGGATCCTGGATCGAGCTGC  1488

Query  121   TACATAAATCCTACCGTATAATATTAGGAGGAGCACAGTTGGAATGTAGAAAGCTCCACA  180
             ||||||||||||||||||||||||||||||||||||||||||||||||||||||||||||
Sbjct  1487  TACATAAATCCTACCGTATAATATTAGGAGGAGCACAGTTGGAATGTAGAAAGCTCCACA  1428

Query  181   GGTGGAATAAATTGTGTAGGAAATCTGATCTGTGTTCACATTACACTTCGCGATTTCTTC  240
             ||||||||||||||||||||||||||||||||||||||||||||||||||||||||||||
Sbjct  1427  GGTGGAATAAATTGTGTAGGAAATCTGATCTGTGTTCACATTACACTTCGCGATTTCTTC  1368

Query  241   ATGAGCTTTCACTTGCCTCCAGAAAAATGGTGGCACAGAAATACTAATGGAGATCATCCA  300
             ||||||||||||||||||||||||||||||||||||||||||||||||||||||||||||
Sbjct  1367  ATGAGCTTTCACTTGCCTCCAGAAAAATGGTGGCACAGAAATACTAATGGAGATCATCCA  1308

Query  301   AACCACAGCGATCATGAGCATTGCTCGGCCAGCAGTCCGGCGTTTGGCATATTCCAAAGC  360
             ||||||||||||||||||||||||||||||||||||||||||||||||||||||||||||
Sbjct  1307  AACCACAGCGATCATGAGCATTGCTCGGCCAGCAGTCCGGCGTTTGGCATATTCCAAAGC  1248

Query  361   ATCTGTGATAGCCCAGTATCTGTCCAGTACAATAACACAGAGGTGTAGGATTGAGGCTGT  420
             |||||||||||||||||||||||||||| |||||||||||||||||||||||||||||||
Sbjct  1247  ATCTGTGATAGCCCAGTATCTGTCCAGTGCAATAACACAGAGGTGTAGGATTGAGGCTGT  1188

Query  421   GCAGCACGTGATGTCTGATGATAACCAGATATCACACAGCACTTGGCCAAAGGCCCACGT  480
             ||||||||||||||||||||||||||||||||||||||||||||||||||||||||||||
Sbjct  1187  GCAGCACGTGATGTCTGATGATAACCAGATATCACACAGCACTTGGCCAAAGGCCCACGT  1128

Query  481   GTGGGTGACAGTGTAAGCGATACTGATGGGCATCACAAGGACAGACACTAAAAGATCTGT  540
             ||||||||||||||||||||||||||||||||||||||||||||||||||||||||||||
Sbjct  1127  GTGGGTGACAGTGTAAGCGATACTGATGGGCATCACAAGGACAGACACTAAAAGATCTGT  1068

Query  541   TACTGCCAAGGAGCCAATGAGGTAATTTGCAGGTGTGTGGAGCTTTCTAGTCaaaaaaaT  600
             ||||||||||||||||||||||||||||||||||||||||||||||||||||||||||||
Sbjct  1067  TACTGCCAAGGAGCCAATGAGGTAATTTGCAGGTGTGTGGAGCTTTCTAGTCAAAAAAAT  1008

Query  601   TGTAATAACAACAAAAACATTTGCAAGGATTGTTGCCAAAGTTATGACAGACAGAAGGAC  660
             ||||||||||||||||||||||||||||||||||||||||||||||||||||||||||||
Sbjct  1007  TGTAATAACAACAAAAACATTTGCAAGGATTGTTGCCAAAGTTATGACAGACAGAAGGAC  948

Query  661   TGACAGGGATATCTTCAGCCCTAACAGTGTCCTTTCATCCACGG  704
              |||||||||||||||||||||||||||||||||||||||||||
Sbjct  947   CGACAGGGATATCTTCAGCCCTAACAGTGTCCTTTCATCCACGG  904
```

5-HTR1E

```
>PREDICTED: Gallus gallus 5-hydroxytryptamine receptor 1E (HTR1E), transcript variant X1, mRNA
Sequence ID: XM_040667699.2 Length: 6400
Range 1: 1428 to 2153

Score:1341 bits(726), Expect:0.0,
Identities:726/726(100%),  Gaps:0/726(0%), Strand: Plus/Plus

Query  1     ACAACCCTGACTATGCTGCTGAATTCTGCTGTCATTGCAGCAATCTCCACAACCAAGAAG  60
             ||||||||||||||||||||||||||||||||||||||||||||||||||||||||||||
Sbjct  1428  ACAACCCTGACTATGCTGCTGAATTCTGCTGTCATTGCAGCAATCTCCACAACCAAGAAG  1487

Query  61    CTTCACCAGCCTGCAAATTATTTAATATGCTCGCTAGCTGTGACAGACCTCCTTGTTGCT  120
             ||||||||||||||||||||||||||||||||||||||||||||||||||||||||||||
Sbjct  1488  CTTCACCAGCCTGCAAATTATTTAATATGCTCGCTAGCTGTGACAGACCTCCTTGTTGCT  1547

Query  121   GTCCTCGTCATGCCCTTGAGTATCACTTACATAATGATAGATAAGTGGACTTTGGGATAC  180
             ||||||||||||||||||||||||||||||||||||||||||||||||||||||||||||
Sbjct  1548  GTCCTCGTCATGCCCTTGAGTATCACTTACATAATGATAGATAAGTGGACTTTGGGATAC  1607

Query  181   TTCATCTGTGAGATATGGTTGAGCGTCGACATGACTTGTTGCACGTGTTCAATTCTTCAC  240
             ||||||||||||||||||||||||||||||||||||||||||||||||||||||||||||
Sbjct  1608  TTCATCTGTGAGATATGGTTGAGCGTCGACATGACTTGTTGCACGTGTTCAATTCTTCAC  1667

Query  241   CTATGTGTTATTGCTCTGGACAGGTACTGGGCAATCACTGATGCCATCGAATACGCCAGG  300
             ||||||||||||||||||||||||||||||||||||||||||||||||||||||||||||
Sbjct  1668  CTATGTGTTATTGCTCTGGACAGGTACTGGGCAATCACTGATGCCATCGAATACGCCAGG  1727

Query  301   AAAAGAACAGCAAAAAGGGCTGGGTTGATGATAGTCACCGTGTGGACTATCTCCATTTTC  360
             ||||||||||||||||||||||||||||||||||||||||||||||||||||||||||||
Sbjct  1728  AAAAGAACAGCAAAAAGGGCTGGGTTGATGATAGTCACCGTGTGGACTATCTCCATTTTC  1787

Query  361   ATATCAATGCCCCCTTTGTTTTGGAGAAACCACCACAGCGTCAGTATTCCCAGTGAGTGC  420
             ||||||||||||||||||||||||||||||||||||||||||||||||||||||||||||
Sbjct  1788  ATATCAATGCCCCCTTTGTTTTGGAGAAACCACCACAGCGTCAGTATTCCCAGTGAGTGC  1847

Query  421   CGCATTCAGCACGACCATGTCATCTACACTATTTATTCCACATTTGGGGCATTTTACATA  480
             ||||||||||||||||||||||||||||||||||||||||||||||||||||||||||||
Sbjct  1848  CGCATTCAGCACGACCATGTCATCTACACTATTTATTCCACATTTGGGGCATTTTACATA  1907

Query  481   CCCTTGACTTTGATCCTGATCCTGTACTACAGAATCTACCACGCTGCAAAGAGTCTCTAC  540
             ||||||||||||||||||||||||||||||||||||||||||||||||||||||||||||
Sbjct  1908  CCCTTGACTTTGATCCTGATCCTGTACTACAGAATCTACCACGCTGCAAAGAGTCTCTAC  1967

Query  541   CAGAAGCGGGGCTCAAGCCGCCATCTCAGCAACAGGAGCACCGACAGCCAAAACTCCTTC  600
             ||||||||||||||||||||||||||||||||||||||||||||||||||||||||||||
Sbjct  1968  CAGAAGCGGGGCTCAAGCCGCCATCTCAGCAACAGGAGCACCGACAGCCAAAACTCCTTC  2027

Query  601   GCCAGCTGCAAGCTCACGCAGACGTTCTGCGTCTCGGACTTCTCCACCTCTGACCCCACC  660
             ||||||||||||||||||||||||||||||||||||||||||||||||||||||||||||
Sbjct  2028  GCCAGCTGCAAGCTCACGCAGACGTTCTGCGTCTCGGACTTCTCCACCTCTGACCCCACC  2087

Query  661   ACAGAGTTTGATAAAATTAATGCATCTGTGAGGATCCCTCCTTTTGAGAATGACTTGGAC  720
             ||||||||||||||||||||||||||||||||||||||||||||||||||||||||||||
Sbjct  2088  ACAGAGTTTGATAAAATTAATGCATCTGTGAGGATCCCTCCTTTTGAGAATGACTTGGAC  2147

Query  721   CTGGCT  726
             ||||||
Sbjct  2148  CTGGCT  2153
```

5-HTR1F

```
>PREDICTED: Gallus gallus 5-hydroxytryptamine receptor 1F (HTR1F), transcript variant X3, mRNA
Sequence ID: XM_046913000.1 Length: 2846
Range 1: 834 to 1563

Score:1343 bits(727), Expect:0.0,
Identities:729/730(99%),  Gaps:0/730(0%), Strand: Plus/Minus

Query  1     ATTCAGATCTGGGGCTTCGTATGCTGATGTTGATTTTATCACAGTCCACCAGTGGATCTG  60
             ||||||||||||||||||||||||||||||||||||||||||||||||||||||||||||
Sbjct  1563  ATTCAGATCTGGGGCTTCGTATGCTGATGTTGATTTTATCACAGTCCACCAGTGGATCTG  1504

Query  61    ATGTCTTCTCTATTGTGCTGGGCATTGAAGTCAATTTGGTGGTTCTCTCACCTGCGTCCA  120
             ||||||||||||||||||||||||||||||||||||||||||||||||||||||||||||
Sbjct  1503  ATGTCTTCTCTATTGTGCTGGGCATTGAAGTCAATTTGGTGGTTCTCTCACCTGCGTCCA  1444

Query  121   AAAGGACTTGTCCATTTACCTCCTCCCTGACTATCCGGCTGACACTTCTTCTGTGAAATG  180
             ||||||||||||||||||||||||||||||||||||||||||||||||||||||||||||
Sbjct  1443  AAAGGACTTGTCCATTTACCTCCTCCCTGACTATCCGGCTGACACTTCTTCTGTGAAATG  1384

Query  181   TCTTTGCTGCTTTGTATATCTTGTAATAAAGGATCAGAATCAAGGCCAGGGGGATATAGA  240
             ||||||||||||||||||||||||||||||||||||||||||||||||||||||||||||
Sbjct  1383  TCTTTGCTGCTTTGTATATCTTGTAATAAAGGATCAGAATCAAGGCCAGGGGGATATAGA  1324

Query  241   AGGCGCCAAATGTAGAGTAAATGGTGGAAACAATGTGGTCATGTTTGATGATGCATTCAT  300
             ||||||||||||||||||||||||||||||||||||||||||||||||||||||||||||
Sbjct  1323  AGGCGCCAAATGTAGAGTAAATGGTGGAAACAATGTGGTCATGTTTGATGATGCATTCAT  1264

Query  301   CATCCCTGCCGGTTGTCTGGTGCCGCCAAAACAAAGGCGGCATGGAGATAAAAATGGATA  360
             ||||||||| ||||||||||||||||||||||||||||||||||||||||||||||||||
Sbjct  1263  CATCCCTGCTGGTTGTCTGGTGCCGCCAAAACAAAGGCGGCATGGAGATAAAAATGGATA  1204

Query  361   TGATCCATACCACTGCAATCATGATACCAGCATGCTTTGGTGTCCTTTTCCGTGCATATT  420
             ||||||||||||||||||||||||||||||||||||||||||||||||||||||||||||
Sbjct  1203  TGATCCATACCACTGCAATCATGATACCAGCATGCTTTGGTGTCCTTTTCCGTGCATATT  1144

Query  421   CCACAGCATCTGTGATTGCTCTGTAACGATCCAAAGCAATGGCAGAGAGATGCAAGATGG  480
             ||||||||||||||||||||||||||||||||||||||||||||||||||||||||||||
Sbjct  1143  CCACAGCATCTGTGATTGCTCTGTAACGATCCAAAGCAATGGCAGAGAGATGCAAGATGG  1084

Query  481   AGCATGTGCAGCATGTAATGTCCACACTCAGCCAAATGTCACACACTACTTGTCCCATGA  540
             ||||||||||||||||||||||||||||||||||||||||||||||||||||||||||||
Sbjct  1083  AGCATGTGCAGCATGTAATGTCCACACTCAGCCAAATGTCACACACTACTTGTCCCATGA  1024

Query  541   TCCAAGTCTCCTTTACAATGTAAACAATGCTGAAGGGCATCACTAGGACTGCCACAAGGA  600
             ||||||||||||||||||||||||||||||||||||||||||||||||||||||||||||
Sbjct  1023  TCCAAGTCTCCTTTACAATGTAAACAATGCTGAAGGGCATCACTAGGACTGCCACAAGGA  964

Query  601   AATCAGTCACTGCAAGAGAGCAGATCAAATAGTTGGCGGGGTGGTGGAGTTTTCTGGTCA  660
             ||||||||||||||||||||||||||||||||||||||||||||||||||||||||||||
Sbjct  963   AATCAGTCACTGCAAGAGAGCAGATCAAATAGTTGGCGGGGTGGTGGAGTTTTCTGGTCA  904

Query  661   CAATTATTGCAGTCATCACCAGAGAATTGATGGCTGTTGTCATTAGCGCAAGCACAGACA  720
             ||||||||||||||||||||||||||||||||||||||||||||||||||||||||||||
Sbjct  903   CAATTATTGCAGTCATCACCAGAGAATTGATGGCTGTTGTCATTAGCGCAAGCACAGACA  844

Query  721   GGGTAATGGA  730
             ||||||||||
Sbjct  843   GGGTAATGGA  834
```

5-HTR2A

```
>PREDICTED: Gallus gallus 5-hydroxytryptamine receptor 2A (HTR2A), transcript variant X1, mRNA
Sequence ID: XM_046908587.1 Length: 3116
Range 1: 1620 to 2350

Score:1351 bits(731), Expect:0.0,
Identities:731/731(100%),  Gaps:0/731(0%), Strand: Plus/Plus

Query  1     GTGTTTAAGAAAGGCCACTGCCTCCTTGCAGATGAGAATTTTGTCCTAATAGGCTCCTTT  60
             ||||||||||||||||||||||||||||||||||||||||||||||||||||||||||||
Sbjct  1620  GTGTTTAAGAAAGGCCACTGCCTCCTTGCAGATGAGAATTTTGTCCTAATAGGCTCCTTT  1679

Query  61    GCAGCATTCTTCATCCCTCTAACCATCATGGTGGTCACTTATTTTTTAACTATCAGGTCC  120
             ||||||||||||||||||||||||||||||||||||||||||||||||||||||||||||
Sbjct  1680  GCAGCATTCTTCATCCCTCTAACCATCATGGTGGTCACTTATTTTTTAACTATCAGGTCC  1739

Query  121   CTGCAGAAAGAAGCCATGCTGTGCGTGAATGACATTGGCCCAAAGACCAAGTTTGCCTCT  180
             ||||||||||||||||||||||||||||||||||||||||||||||||||||||||||||
Sbjct  1740  CTGCAGAAAGAAGCCATGCTGTGCGTGAATGACATTGGCCCAAAGACCAAGTTTGCCTCT  1799

Query  181   TTTAGTTTCCTCCCTCAGAGCTCCCTTTCTTCAGAGAAACTCTTCCAGCGCTCTTTGAAC  240
             ||||||||||||||||||||||||||||||||||||||||||||||||||||||||||||
Sbjct  1800  TTTAGTTTCCTCCCTCAGAGCTCCCTTTCTTCAGAGAAACTCTTCCAGCGCTCTTTGAAC  1859

Query  241   AGGGACATGGGGACCTCCGGGAGGAGAACCATGCAATCCATCAGCAATGAGCAGAAAGCT  300
             ||||||||||||||||||||||||||||||||||||||||||||||||||||||||||||
Sbjct  1860  AGGGACATGGGGACCTCCGGGAGGAGAACCATGCAATCCATCAGCAATGAGCAGAAAGCT  1919

Query  301   TCCAAGGTCCTTGGCATTGTCTTCTTTCTGTTTGTTGTGATGTGGTGCCCGTTTTTCATC  360
             ||||||||||||||||||||||||||||||||||||||||||||||||||||||||||||
Sbjct  1920  TCCAAGGTCCTTGGCATTGTCTTCTTTCTGTTTGTTGTGATGTGGTGCCCGTTTTTCATC  1979

Query  361   ACCAATGTGATGGCTGTAATTTGTAAGGAGTCATGCAACGAAGAAGTCATTGGTGGGCTA  420
             ||||||||||||||||||||||||||||||||||||||||||||||||||||||||||||
Sbjct  1980  ACCAATGTGATGGCTGTAATTTGTAAGGAGTCATGCAACGAAGAAGTCATTGGTGGGCTA  2039

Query  421   CTTAACATATTTGTTTGGATTGGGTACCTTTCTTCAGCCGTCAATCCACTCGTATATACG  480
             ||||||||||||||||||||||||||||||||||||||||||||||||||||||||||||
Sbjct  2040  CTTAACATATTTGTTTGGATTGGGTACCTTTCTTCAGCCGTCAATCCACTCGTATATACG  2099

Query  481   TTGTTCAATAAGACCTACCGCTCAGCTTTCTCTCGCTACATTCAGTGTCGCTACAAGGAG  540
             ||||||||||||||||||||||||||||||||||||||||||||||||||||||||||||
Sbjct  2100  TTGTTCAATAAGACCTACCGCTCAGCTTTCTCTCGCTACATTCAGTGTCGCTACAAGGAG  2159

Query  541   GAGAAGAAACCTTTCCAGCTGATTTTAGTGAATACTATCCCAGCGCTTGCGTATAATTCT  600
             ||||||||||||||||||||||||||||||||||||||||||||||||||||||||||||
Sbjct  2160  GAGAAGAAACCTTTCCAGCTGATTTTAGTGAATACTATCCCAGCGCTTGCGTATAATTCT  2219

Query  601   AGCCAGCTCCACCTGGCTCAAATGAAGAGCTTGAAAAAAGAGGCAAAATTGATGAGTAAG  660
             ||||||||||||||||||||||||||||||||||||||||||||||||||||||||||||
Sbjct  2220  AGCCAGCTCCACCTGGCTCAAATGAAGAGCTTGAAAAAAGAGGCAAAATTGATGAGTAAG  2279

Query  661   GATTATTCGATGGTCACGATAGGAATACGCCATATGGATGGTACCTCAAAGGGAAGCATC  720
             ||||||||||||||||||||||||||||||||||||||||||||||||||||||||||||
Sbjct  2280  GATTATTCGATGGTCACGATAGGAATACGCCATATGGATGGTACCTCAAAGGGAAGCATC  2339

Query  721   AGTCCAGGGAA  731
             |||||||||||
Sbjct  2340  AGTCCAGGGAA  2350
```

5-HTR2B

```
>Gallus gallus 5-hydroxytryptamine receptor 2B (HTR2B), transcript variant 2, mRNA
Sequence ID: NM_001397883.1 Length: 2670
Range 1: 1048 to 1807

Score:1393 bits(754), Expect:0.0,
Identities:758/760(99%),  Gaps:0/760(0%), Strand: Plus/Minus

Query  1     CTTTTCTGGTGAGCAGGCAGGTGTGGCGTCGCGCTGAAATACTGTGGACACTGTCGACCA  60
             ||||||||||||||||||||||||||||||||||||||||||||||||||||||||||||
Sbjct  1807  CTTTTCTGGTGAGCAGGCAGGTGTGGCGTCGCGCTGAAATACTGTGGACACTGTCGACCA  1748

Query  61    AGTGAAACGTTGAGGTGGCTTGTTGATCAAATAGGCCTTCTTGCGTAGCACTTGAATTGT  120
             ||||||||||||||||||||||||||||||||||||||||||||||||||||||||||||
Sbjct  1747  AGTGAAACGTTGAGGTGGCTTGTTGATCAAATAGGCCTTCTTGCGTAGCACTTGAATTGT  1688

Query  121   CAGAAAGTAAGTGACTATCATGATAGCAAGGGGAATGAAGAACGCAGCCACTGATCCATA  180
             ||||||||||||||||||||||||||||||||||||||||||||||||||||||||||||
Sbjct  1687  CAGAAAGTAAGTGACTATCATGATAGCAAGGGGAATGAAGAACGCAGCCACTGATCCATA  1628

Query  181   GAGAATGAAGTCATGAAAGCGATCAGGCGTCAGAAGACACGTGATATTTGTAGAGTTGCC  240
             ||||||||||||||||||||||||||||||||||||||||||||||||||||||||||||
Sbjct  1627  GAGAATGAAGTCATGAAAGCGATCAGGCGTCAGAAGACACGTGATATTTGTAGAGTTGCC  1568

Query  241   ATTTCCATCTTCAATTCCTCTGATAGGGACTGGAATAGCAATGCCTATTGAAATGAGCCA  300
             ||||||||||||||||||||||||||||||||||||||||||||||||||||||||||||
Sbjct  1567  ATTTCCATCTTCAATTCCTCTGATAGGGACTGGAATAGCAATGCCTATTGAAATGAGCCA  1508

Query  301   AACAATGATGATTTTGATGATTGTTGTAGCCCATGAGTTGTACTGGCTGGCCTGGATTGG  360
             ||||||||||||||||||||||||||||||||||||||||||||||||||||||||||||
Sbjct  1507  AACAATGATGATTTTGATGATTGTTGTAGCCCATGAGTTGTACTGGCTGGCCTGGATTGG  1448

Query  361   CTTTTTAATGGCAATATAGCGGTCTAGTGAGATGGCACAGAGGTGCATGATGGAAGCTGT  420
             ||||||||||||||||||||||||||||||||||||||||||||||||||||||||||||
Sbjct  1447  CTTTTTAATGGCAATATAGCGGTCTAGTGAGATGGCACAGAGGTGCATGATGGAAGCTGT  1388

Query  421   GGAGAAGAGGACATCAAGGAGCAGCCAAATGGGACACAAGGCAGTTGGAAAAGGCCAGGC  480
             ||||||||||||||||||| ||||||||||||||||||||||||||||||||||||||||
Sbjct  1387  GGAGAAGAGGACATCAAGGAACAGCCAAATGGGACACAAGGCAGTTGGAAAAGGCCAGGC  1328

Query  481   GTTGTCAAAGAGTATTATGAGAAGGGCAATCGGCATCACAAACAGCCCCACGAGCAAATC  540
             ||||||||||||||||||||||||||||||||||||||||||||||||||||||||||||
Sbjct  1327  GTTGTCAAAGAGTATTATGAGAAGGGCAATCGGCATCACAAACAGCCCCACGAGCAAATC  1268

Query  541   TGCCACCGCCAGGGATGTTAAAAAGTAATTGGTGGCATATTGCAACTTTTTCTCCAGAGA  600
             ||||||||||||||||||||||||||| ||||||||||||||||||||||||||||||||
Sbjct  1267  TGCCACCGCCAGGGATGTTAAAAAGTAGTTGGTGGCATATTGCAACTTTTTCTCCAGAGA  1208

Query  601   TACCGCCAGGATGACCAGGATGTTCCCACCGATGGTGGGGATGATCACCAGCAGGATCAG  660
             ||||||||||||||||||||||||||||||||||||||||||||||||||||||||||||
Sbjct  1207  TACCGCCAGGATGACCAGGATGTTCCCACCGATGGTGGGGATGATCACCAGCAGGATCAG  1148

Query  661   CAGCGCTGCCCAGCGCACTTTGTTTCCTTGCTCATCGTTTGGGTAGTTCTCTTTGGGTTC  720
             ||||||||||||||||||||||||||||||||||||||||||||||||||||||||||||
Sbjct  1147  CAGCGCTGCCCAGCGCACTTTGTTTCCTTGCTCATCGTTTGGGTAGTTCTCTTTGGGTTC  1088

Query  721   TGCTACCGAGAGAGGTGACAAAGATCCATTGCCAGCTCCA  760
             ||||||||||||||||||||||||||||||||||||||||
Sbjct  1087  TGCTACCGAGAGAGGTGACAAAGATCCATTGCCAGCTCCA  1048
```

5-HTR2C

```
>PREDICTED: Gallus gallus 5-hydroxytryptamine receptor 2C (HTR2C), transcript variant X10, mRNA
Sequence ID: XM_046916516.1 Length: 4057
Range 1: 523 to 1283

Score:1400 bits(758), Expect:0.0,
Identities:760/761(99%),  Gaps:0/761(0%), Strand: Plus/Minus

Query  1     TCCATTGACCAACGCTTACATTTTTATACAATATATTTTGGCCACAGATAGCTCGTAGCC  60
             ||||||||||||||||||||||||||||||||||||||||||||||||||||||||||||
Sbjct  1283  TCCATTGACCAACGCTTACATTTTTATACAATATATTTTGGCCACAGATAGCTCGTAGCC  1224

Query  61    AAAGGCTGCGTGAGCCGCCTTTACACACAGCTCACTCTCTCGCTCACAACATTACAGCTG  120
             ||||||||||||||||||||||||||||||||||||||||||||||||||||||||||||
Sbjct  1223  AAAGGCTGCGTGAGCCGCCTTTACACACAGCTCACTCTCTCGCTCACAACATTACAGCTG  1164

Query  121   TTTATGGAGAGCTCTGAAGTCCCTGGTTGCATTTCAAGGCCCTCCTCAGCTTCATCTAGT  180
             ||||||||||||||||||||||||||||||||||||||||||||||||||||||||||||
Sbjct  1163  TTTATGGAGAGCTCTGAAGTCCCTGGTTGCATTTCAAGGCCCTCCTCAGCTTCATCTAGT  1104

Query  181   TCCATGCTGTTGAGTTCATTGCCATTTCGATAACTAGTTAAAGTCAGATCCTTCCCGTAT  240
             ||||||||||||||||||||||||||||||||||||||||||||||||||||||||||||
Sbjct  1103  TCCATGCTGTTGAGTTCATTGCCATTTCGATAACTAGTTAAAGTCAGATCCTTCCCGTAT  1044

Query  241   AAAGCTGTTGAAGCAACATTCAGACACTGATTCTGTCGCAGTGCTGATTTTTTGCTGGTC  300
             ||||||||||||||||||||||||||||||||||||||||||||||||||||||||||||
Sbjct  1043  AAAGCTGTTGAAGCAACATTCAGACACTGATTCTGTCGCAGTGCTGATTTTTTGCTGGTC  984

Query  301   TTGTATTGGCAGCGGATATAACTGGAGAAAGCCCTGCGATAGGTTTTATTGAAGAGTGTG  360
             ||||||||||||||||||||||||||||||||||||||||||||||||||||||||||||
Sbjct  983   TTGTATTGGCAGCGGATATAACTGGAGAAAGCCCTGCGATAGGTTTTATTGAAGAGTGTG  924

Query  361   TATACGAGGGGATTAACCCCTGAGCAAACATACCCCACCCAAACAAACACGTCGAGGAGT  420
             ||||||||||||||||||||||||||||||||||||||||||||||||||||||||||||
Sbjct  923   TATACGAGGGGATTAACCCCTGAGCAAACATACCCCACCCAAACAAACACGTCGAGGAGT  864

Query  421   TCGCTCAACAGGTCTTTATCGCAGGCTTCCTTGCAAAGGACGGACATGACGTTAGTGATG  480
             |||||||| |||||||||||||||||||||||||||||||||||||||||||||||||||
Sbjct  863   TCGCTCAAGAGGTCTTTATCGCAGGCTTCCTTGCAAAGGACGGACATGACGTTAGTGATG  804

Query  481   AAAAATGGGCACCACATGATGAGAAACAAAAAGAAGACGATGCCCAGGACCTTGGAGGCT  540
             ||||||||||||||||||||||||||||||||||||||||||||||||||||||||||||
Sbjct  803   AAAAATGGGCACCACATGATGAGAAACAAAAAGAAGACGATGCCCAGGACCTTGGAGGCT  744

Query  541   CTTCGCTCGTTGTTGATGGACTGCATAGTTCCTTTCCGAAAAAGCACTGCCTCCTTACTT  600
             ||||||||||||||||||||||||||||||||||||||||||||||||||||||||||||
Sbjct  743   CTTCGCTCGTTGTTGATGGACTGCATAGTTCCTTTCCGAAAAAGCACTGCCTCCTTACTT  684

Query  601   ACAGGAGAGTTCAAATGCGATGCCCCCTCGTGATTGTGAAGCATCGAGATGTGCTCTGTG  660
             ||||||||||||||||||||||||||||||||||||||||||||||||||||||||||||
Sbjct  683   ACAGGAGAGTTCAAATGCGATGCCCCCTCGTGATTGTGAAGCATCGAGATGTGCTCTGTG  624

Query  661   TTGTTTTCTTTCTTGAGGCAGTTCACGCTGCTCCTCCTCTGCTTGGGCACCTCTCCACAC  720
             ||||||||||||||||||||||||||||||||||||||||||||||||||||||||||||
Sbjct  623   TTGTTTTCTTTCTTGAGGCAGTTCACGCTGCTCCTCCTCTGCTTGGGCACCTCTCCACAC  564

Query  721   ATGAACACCGTGGCCTGTCTCTGTAGCACCTGGACAGTCAG  761
             |||||||||||||||||||||||||||||||||||||||||
Sbjct  563   ATGAACACCGTGGCCTGTCTCTGTAGCACCTGGACAGTCAG  523
```

5-HTR3A

```
>PREDICTED: Gallus gallus 5-hydroxytryptamine receptor 3A (HTR3A), transcript variant X2, partial mRNA
Sequence ID: XM_040690408.2 Length: 7364
Range 1: 335 to 1070

Score:1321 bits(715), Expect:0.0,
Identities:729/736(99%),  Gaps:0/736(0%), Strand: Plus/Minus

Query  1     CCGTAGTGGTTCAGTTTGGCTGTGCTGTCATTGTTCTCCACGTGCCTGGAGATGTCTGAG  60
             ||||||||||||||||||||||||||||||||||||||||||||||||||||||||||||
Sbjct  1070  CCGTAGTGGTTCAGTTTGGCTGTGCTGTCATTGTTCTCCACGTGCCTGGAGATGTCTGAG  1011

Query  61    CTTTGTGTTCTGCTTTGGCTGAATTTCTTCCTGTCCCAGATGCAGAGCAGTGCAATGGCA  120
             || ||||||||||||||||||||||||||||||||||||||||||||||||||| |||||
Sbjct  1010  CTCTGTGTTCTGCTTTGGCTGAATTTCTTCCTGTCCCAGATGCAGAGCAGTGCAGTGGCA  951

Query  121   CGTTCCAGCAGTAAGTGCTTCACCCAGCTGGGGACGTGAGGCTGCAGGTCTTGCTTGTGT  180
             ||||||||||||||||||||||||||| ||||||||||||||||||||||||||||||||
Sbjct  950   CGTTCCAGCAGTAAGTGCTTCACCCAGATGGGGACGTGAGGCTGCAGGTCTTGCTTGTGT  891

Query  181   ACCAGGCGCACAATGAAGATGGTCTCTGTGAGGCTGTTGACGAGCAACGCCATGCACACC  240
             |||||||||||||||||||||||||||||||||||| |||||||||||||||||||||||
Sbjct  890   ACCAGGCGCACAATGAAGATGGTCTCTGTGAGGCTGATGACGAGCAACGCCATGCACACC  831

Query  241   ACAAAATAGATGCCTATCAGTGGGGTGCCAACAGCAGTAGCTGGAAGCGTGTCAGATACG  300
             ||||||||||||||||||||||||||||||||||||||||||||||||||||||||||||
Sbjct  830   ACAAAATAGATGCCTATCAGTGGGGTGCCAACAGCAGTAGCTGGAAGCGTGTCAGATACG  771

Query  301   ATGATGAGGAAAACTGAGTAGCCCAGGAGGAGAGTGATCTTGAAAGAGACCCTCTCACCA  360
             |||||||||||||||||||||||||||||||||||||||||||||||||||||||| |||
Sbjct  770   ATGATGAGGAAAACTGAGTAGCCCAGGAGGAGAGTGATCTTGAAAGAGACCCTCTCGCCA  711

Query  361   CTGTTGGGAGGCAGGTAGAAGCCTACAATGTCCATCACCATCAAGAAGATACTAGGGAGC  420
             ||||||||||||||||||||||||||||||||||||||||||||||||||||||||||||
Sbjct  710   CTGTTGGGAGGCAGGTAGAAGCCTACAATGTCCATCACCATCAAGAAGATACTAGGGAGC  651

Query  421   AGCAGGTTGATGGTATAGAAGAGAGGACGCCTCCGGATGACAACGTAGAACTTCATCTCG  480
             ||||||||||||||||||||||||||||||||||||||||| ||||||||||||||||||
Sbjct  650   AGCAGGTTGATGGTATAGAAGAGAGGACGCCTCCGGATGACGACGTAGAACTTCATCTCG  591

Query  481   GCGTAGCTGTCGCTGCTTTTGACGCTGAACTCCTGGAAGTGGCTGAGCACGTACAGCAGC  540
             ||||||||||||||||||||||||||||||||||||||||||||||||||||||||||||
Sbjct  590   GCGTAGCTGTCGCTGCTTTTGACGCTGAACTCCTGGAAGTGGCTGAGCACGTACAGCAGC  531

Query  541   TCCCACTCGCCCTGGCTCATGAAGACGCTCCTGTCGAATTTCACCAGCTCCGGCTGCCGC  600
             ||||||||||||||| ||||||||||||||||||||||||||||||||||||||||||||
Sbjct  530   TCCCACTCGCCCTGGTTCATGAAGACGCTCCTGTCGAATTTCACCAGCTCCGGCTGCCGC  471

Query  601   CACAGCGAGAGGTTGATGTCGTGGATGTTGTGCAGCCAGCTGGTGAAGGTAAGCGAGCAG  660
             ||||||||||||||||||||||||||||||||||||||||||||||||||||||||||||
Sbjct  470   CACAGCGAGAGGTTGATGTCGTGGATGTTGTGCAGCCAGCTGGTGAAGGTAAGCGAGCAG  411

Query  661   TTCTGAACGTCGAAGGGGAAATTGTAGATGTCCAAGCTGCAGGCTGTCACCACTTGGATG  720
             ||||||||||||||||||||||||||||||||||||||||||||||||||||||||||||
Sbjct  410   TTCTGAACGTCGAAGGGGAAATTGTAGATGTCCAAGCTGCAGGCTGTCACCACTTGGATG  351

Query  721   GGCTTGAGGTTCTGGA  736
             ||||||||||||||||
Sbjct  350   GGCTTGAGGTTCTGGA  335
```

5-HTR4

```
>PREDICTED: Gallus gallus 5-hydroxytryptamine receptor 4 (HTR4), transcript variant X5, mRNA
Sequence ID: XM_040682639.2 Length: 8603
Range 1: 234 to 990

Score:1376 bits(745), Expect:0.0,
Identities:753/757(99%),  Gaps:0/757(0%), Strand: Plus/Minus

Query  1    GTCTTGGCAGCTTTGGTCTCGGTCTTCATGCGATGAGTGCTGTGCTGGTCAGGCTGGTGC  60
            ||||||||||||||||||||||||||||||||||| ||||||||||||||||||||||||
Sbjct  990  GTCTTGGCAGCTTTGGTCTCGGTCTTCATGCGATGCGTGCTGTGCTGGTCAGGCTGGTGC  931

Query  61   CTGCTGTCAGCAGGGGTCCCTGCGCGCTGCAGCACCCGGATCTGCCGGGCGTGCTCCCGG  120
            ||||||||||||||||||||||||||||||||||||||||||||||||||||||||||||
Sbjct  930  CTGCTGTCAGCAGGGGTCCCTGCGCGCTGCAGCACCCGGATCTGCCGGGCGTGCTCCCGG  871

Query  121  GCGGTGACGTAGATGCGATAATAGGCCAGCACCATCAGCAGGAAGGGAAAGTAGAAGGCC  180
            ||||||||||||||||||||||||||||||||||||||||||||||||||||||||||||
Sbjct  870  GCGGTGACGTAGATGCGATAATAGGCCAGCACCATCAGCAGGAAGGGAAAGTAGAAGGCC  811

Query  181  ACCACAGAGCAGGTAATGGCATATGGCTTGTTGACCATGAATATGCAGTACGTGGAGTTG  240
            ||||||||||||||||||||||||||||||||||||||||||||||||||||||||||||
Sbjct  810  ACCACAGAGCAGGTAATGGCATATGGCTTGTTGACCATGAATATGCAGTACGTGGAGTTG  751

Query  241  GAAGCCTTGTTGAACTGCCTTTGCTGAATCAAATCAATGATGCCAATGCTATTCCAGCCT  300
            ||||||||||||||||||||||||||||||||||||||||||||||||||||||||||||
Sbjct  750  GAAGCCTTGTTGAACTGCCTTTGCTGAATCAAATCAATGATGCCAATGCTATTCCAGCCT  691

Query  301  TGCATGATAGGGAGGAAAGAAATAAATGTCGGGATTACCCAGCAGCCTCCAAGCATTACA  360
            ||||||||||||||||||||||||||||||||||||||||||||||||||||||||||||
Sbjct  690  TGCATGATAGGGAGGAAAGAAATAAATGTCGGGATTACCCAGCAGCCTCCAAGCATTACA  631

Query  361  GCAATACGCAGCGGAGTCATCTTGTTCCTGTACACCAGTGGCTGGCAACAGATAGCGTAG  420
            ||||||||||| ||||||||||||||||||||||||||||||||||||||||||||||||
Sbjct  630  GCAATACGCAGTGGAGTCATCTTGTTCCTGTACACCAGTGGCTGGCAACAGATAGCGTAG  571

Query  421  TACCTGTCCAGTGATATGCAGCACAGGTGCAGGATCGATGCTGTGGTGAGCAAGACATCG  480
            ||||||||||||||||||||||||||||||||||||||||||||||||||||||||||||
Sbjct  570  TACCTGTCCAGTGATATGCAGCACAGGTGCAGGATCGATGCTGTGGTGAGCAAGACATCG  511

Query  481  AGGGATGTTCGGACGAGGCAGAACATCTCCCCATAGATCCAGTTGTCCTGGACCAACTCA  540
            ||||||||||||||||||||||||||||||||||||||||||||||||||||||||||||
Sbjct  510  AGGGATGTTCGGACGAGGCAGAACATCTCCCCATAGATCCAGTTGTCCTGGACCAACTCA  451

Query  541  ATGGCTCCAAACGGCATCACCAGTACTGACACCAGCAGGTCCGCGAAGGCAAGAGAAACG  600
            ||||||||||||||||||||||||||||||||||||||||||||||||||||||||||||
Sbjct  450  ATGGCTCCAAACGGCATCACCAGTACTGACACCAGCAGGTCCGCGAAGGCAAGAGAAACG  391

Query  601  ATGAAATAATTGGTCTTGATTTTCCTGAGCTGCCTGTCCCGGCACACAGCCACCATCACC  660
            ||||||||||||||||||||||||||||||||||||||||||||||||||||||||||||
Sbjct  390  ATGAAATAATTGGTCTTGATTTTCCTGAGCTGCCTGTCCCGGCACACAGCCACCATCACC  331

Query  661  AACAGGTTCCCCAGGATGGCCATCAGGATAACTGCGGAGATGAAGGTGAGCAGTACGATC  720
            ||||||||||||||||||||||||||||||||||| ||||||||||||||||||||||||
Sbjct  330  AACAGGTTCCCCAGGATGGCCATCAGGATAACTGCAGAGATGAAGGTGAGCAGTACGATC  271

Query  721  TTCTCTGCTACGCCGAAGCCCTCACTCGAACTCCCAT  757
            ||||||||||||||||||||||||||||||||| |||
Sbjct  270  TTCTCTGCTACGCCGAAGCCCTCACTCGAACTCACAT  234
```

5-HTR5A

```
>PREDICTED: Gallus gallus 5-hydroxytryptamine receptor 5A (HTR5A), mRNA
Sequence ID: XM_040665750.2 Length: 4907
Range 1: 169 to 900

Score:1347 bits(729), Expect:0.0,
Identities:731/732(99%),  Gaps:0/732(0%), Strand: Plus/Plus

Query  1    AGTAATCGGAGCGGGTCATCCAGCGGGCTGGAGGGTGGCAGGGCACAGCTCTCCGTCTTC  60
            ||||||||||||||||||||||||||||||||||||||||||||||||||||||||||||
Sbjct  169  AGTAATCGGAGCGGGTCATCCAGCGGGCTGGAGGGTGGCAGGGCACAGCTCTCCGTCTTC  228

Query  61   AGTGTTCTTGTCCTCACCCTCCTGGCCATGCTGGTGGTGGCCACTTTCCTGTGGAACGGC  120
            ||||||||||||||||||||||||||||||||||||||||||||||||||||||||||||
Sbjct  229  AGTGTTCTTGTCCTCACCCTCCTGGCCATGCTGGTGGTGGCCACTTTCCTGTGGAACGGC  288

Query  121  TTGGTCCTGGCCACCATCTTCCGCGTGCGCACGTTTCATCGGGTGCCTCACAACCTGGTG  180
            ||||||||||||||||||||||||||||||||||||||||||||||||||||||||||||
Sbjct  289  TTGGTCCTGGCCACCATCTTCCGCGTGCGCACGTTTCATCGGGTGCCTCACAACCTGGTG  348

Query  181  GCATCCATGGCCATCTCCGACGTGATGGTGGCCGCCCTCGTCATGCCCCTGAGCTTGGTG  240
            ||||||||||||||||||||||||||||||||||||||||||||||||||||||||||||
Sbjct  349  GCATCCATGGCCATCTCCGACGTGATGGTGGCCGCCCTCGTCATGCCCCTGAGCTTGGTG  408

Query  241  CACGAGTTGTCGGGGCGGAGGTGGCGGCTGGGTCGGTCTCTGTGCCAGGTGTGGATCTCC  300
            ||||||||||||||||||||||||||||||||||||||||||||||||||||||||||||
Sbjct  409  CACGAGTTGTCGGGGCGGAGGTGGCGGCTGGGTCGGTCTCTGTGCCAGGTGTGGATCTCC  468

Query  301  TTCGATGTCCTGTGCTGCACTGCCAGCATCTGGAACGTCACTGCCATCGCCCTCGACCGC  360
            ||||||||||||||||||||||||||||||||||||||||||||||||||||||||||||
Sbjct  469  TTCGATGTCCTGTGCTGCACTGCCAGCATCTGGAACGTCACTGCCATCGCCCTCGACCGC  528

Query  361  TACTGGTCCATCACCCGGCACCTGGAATACACGCTGCGCACCCGACGCCGTATCTCCAAC  420
            |||||||||||||||||||||||||| |||||||||||||||||||||||||||||||||
Sbjct  529  TACTGGTCCATCACCCGGCACCTGGAGTACACGCTGCGCACCCGACGCCGTATCTCCAAC  588

Query  421  ATCATGATTGCGCTCACCTGGGTGCTCTCTGCCTTCATCTCTCTGGCCCCGCTGCTCTTT  480
            ||||||||||||||||||||||||||||||||||||||||||||||||||||||||||||
Sbjct  589  ATCATGATTGCGCTCACCTGGGTGCTCTCTGCCTTCATCTCTCTGGCCCCGCTGCTCTTT  648

Query  481  GGCTGGGGAGAGACTTATTCAGAGGACAGTGAGGAGTGCCAGGTAAGCCAGGAGCCTTCC  540
            ||||||||||||||||||||||||||||||||||||||||||||||||||||||||||||
Sbjct  649  GGCTGGGGAGAGACTTATTCAGAGGACAGTGAGGAGTGCCAGGTAAGCCAGGAGCCTTCC  708

Query  541  TACACCATCTTCTCCACCTTCGGGGCCTTCTACCTGCCCCTATGTGTGGTGCTGTTTGTC  600
            ||||||||||||||||||||||||||||||||||||||||||||||||||||||||||||
Sbjct  709  TACACCATCTTCTCCACCTTCGGGGCCTTCTACCTGCCCCTATGTGTGGTGCTGTTTGTC  768

Query  601  TACTGGAAGATCTACAAGGCGGCCAAGTTTCGCATCGGATCTCGGAAGAGCAACTCCATC  660
            ||||||||||||||||||||||||||||||||||||||||||||||||||||||||||||
Sbjct  769  TACTGGAAGATCTACAAGGCGGCCAAGTTTCGCATCGGATCTCGGAAGAGCAACTCCATC  828

Query  661  ACCCCCGTCTCTCCAGAAGCCCCAGAGATAAAGGAAGCTGCCCAGCAGCCACAGATGGTC  720
            ||||||||||||||||||||||||||||||||||||||||||||||||||||||||||||
Sbjct  829  ACCCCCGTCTCTCCAGAAGCCCCAGAGATAAAGGAAGCTGCCCAGCAGCCACAGATGGTC  888

Query  721  TTCACTGTCCGG  732
            ||||||||||||
Sbjct  889  TTCACTGTCCGG  900
```

5-HTR7

```
>Gallus gallus 5-hydroxytryptamine receptor 7 precursor (HTR7) mRNA, complete cds
Sequence ID: GU574812.1 Length: 1396
Range 1: 24 to 623

Score:1098 bits(594), Expect:0.0,
Identities:598/600(99%),  Gaps:0/600(0%), Strand: Plus/Plus

Query  1    CAACGGCAGCCACCTCTACGGTAACCTCCGGCCCTTCGCGCTGGAGGAGCCGCGGGCGCT  60
            ||||||||||||||||||||||||||||||||||||||||||||||||||||||||||||
Sbjct  24   CAACGGCAGCCACCTCTACGGTAACCTCCGGCCCTTCGCGCTGGAGGAGCCGCGGGCGCT  83

Query  61   GAGCGGAGGGAGGATGATCGCCGGCTCGTGGCCCCCGCGCAGCCTGGCCAAGCTCGGCCC  120
            | |||| |||||||||||||||||||||||||||||||||||||||||||||||||||||
Sbjct  84   GGGCGGCGGGAGGATGATCGCCGGCTCGTGGCCCCCGCGCAGCCTGGCCAAGCTCGGCCC  143

Query  121  GGGAGGCACTCCCCCTCCGCTGCCCGCCGCCAGCCCCGCGCCCGGCAACGACTCGCAATG  180
            ||||||||||||||||||||||||||||||||||||||||||||||||||||||||||||
Sbjct  144  GGGAGGCACTCCCCCTCCGCTGCCCGCCGCCAGCCCCGCGCCCGGCAACGACTCGCAATG  203

Query  181  CGGGGAGCAGATCCTCAGCTACGGCAACGTGGAGAAAGTTGTCATCGGGGCCGTGCTCAG  240
            ||||||||||||||||||||||||||||||||||||||||||||||||||||||||||||
Sbjct  204  CGGGGAGCAGATCCTCAGCTACGGCAACGTGGAGAAAGTTGTCATCGGGGCCGTGCTCAG  263

Query  241  CCTCATCACGCTGCTGACTGTCGCCGGCAACTGCCTGGTGGTCATCTCCGTCTGCTTCGT  300
            ||||||||||||||||||||||||||||||||||||||||||||||||||||||||||||
Sbjct  264  CCTCATCACGCTGCTGACTGTCGCCGGCAACTGCCTGGTGGTCATCTCCGTCTGCTTCGT  323

Query  301  GAAGAAGCTGCGGCAGCCCTCCAACTATCTCATCGTCTCTCTGGCCCTGGCCGACCTCTC  360
            ||||||||||||||||||||||||||||||||||||||||||||||||||||||||||||
Sbjct  324  GAAGAAGCTGCGGCAGCCCTCCAACTATCTCATCGTCTCTCTGGCCCTGGCCGACCTCTC  383

Query  361  TGTGGCCCTGGCCGTCATGCCCTTCGTCAGTGTGACTGATCTCATCGGTGGCGAGTGGAT  420
            ||||||||||||||||||||||||||||||||||||||||||||||||||||||||||||
Sbjct  384  TGTGGCCCTGGCCGTCATGCCCTTCGTCAGTGTGACTGATCTCATCGGTGGCGAGTGGAT  443

Query  421  CTTCGGGCGCCTTTTCTGCAACGTCTTCATCGCCATGGACGTCATGTGCTGCACGGCCTC  480
            ||||||||||||||||||||||||||||||||||||||||||||||||||||||||||||
Sbjct  444  CTTCGGGCGCCTTTTCTGCAACGTCTTCATCGCCATGGACGTCATGTGCTGCACGGCCTC  503

Query  481  CATCATGACCCTCTGCGTGATCAGCATCGACAGGTACCTTGGAATAACAAGACCGCTCAC  540
            ||||||||||||||||||||||||||||||||||||||||||||||||||||||||||||
Sbjct  504  CATCATGACCCTCTGCGTGATCAGCATCGACAGGTACCTTGGAATAACAAGACCGCTCAC  563

Query  541  ATATCCTGTGAGGCAGAACGGGAAGTGTATGGCCAAAATGATCCTGTGTGTGTGGCTCTT  600
            ||||||||||||||||||||||||||||||||||||||||||||||||||||||||||||
Sbjct  564  ATATCCTGTGAGGCAGAACGGGAAGTGTATGGCCAAAATGATCCTGTGTGTGTGGCTCTT  623
```

Supplemental Figure 2. Validation of *5-HTR* subfamily RNA probes used in this study

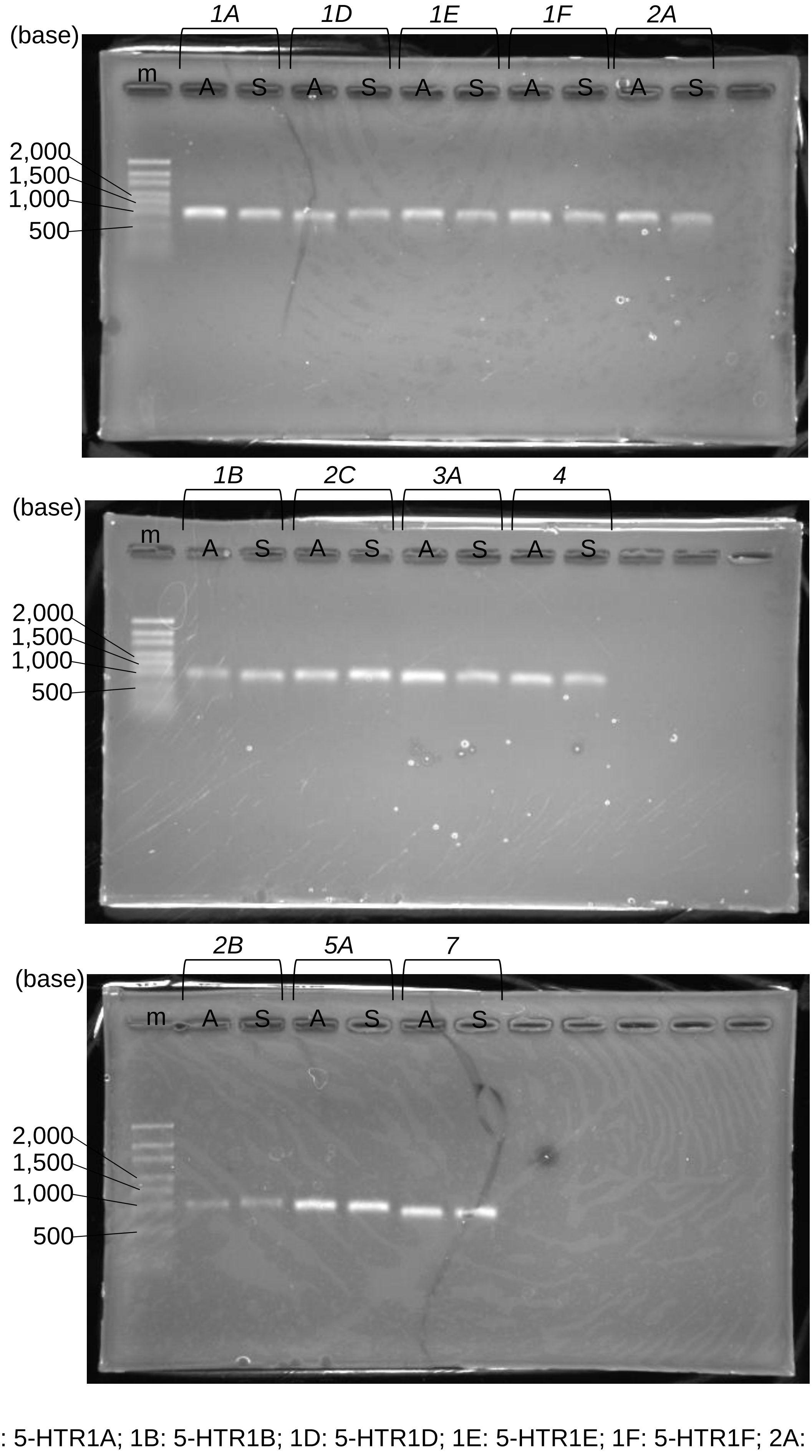

1A: 5-HTR1A; 1B: 5-HTR1B; 1D: 5-HTR1D; 1E: 5-HTR1E; 1F: 5-HTR1F; 2A: 5-HTR2A; 2B: 5-HTR2B; 2C: 5-HTR2C; 3A: 5-HTR3A; 4: 5-HTR4; 5A: 5-HTR5A; 7: 5-HTR7; A: antisense probe; S: sense probe; m: molecular marker.

Supplemental Figure 3. *5-HTR*s expressions in the chick brainstem

Chick #11

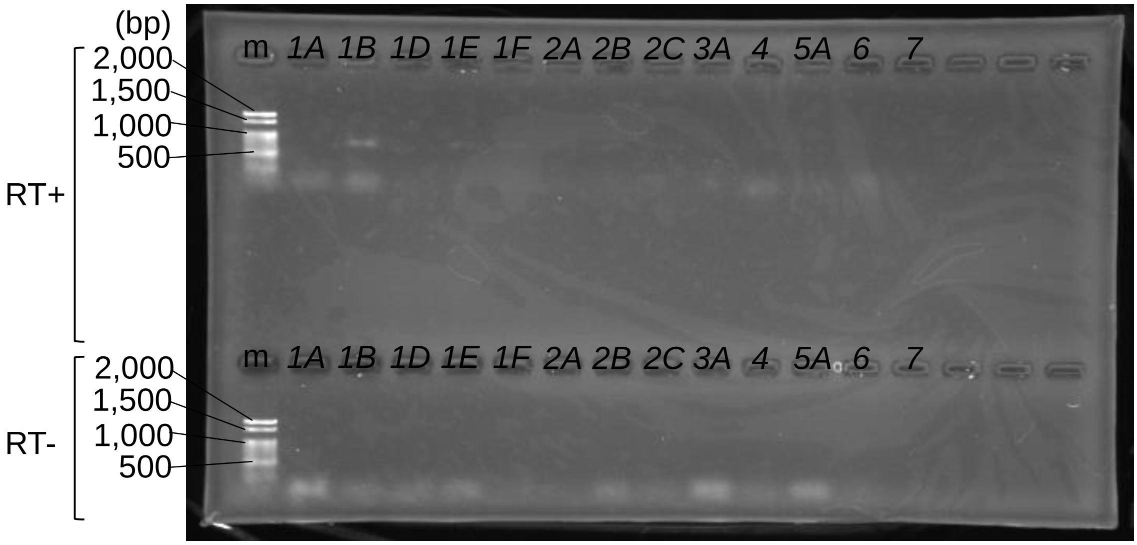

Chick #12

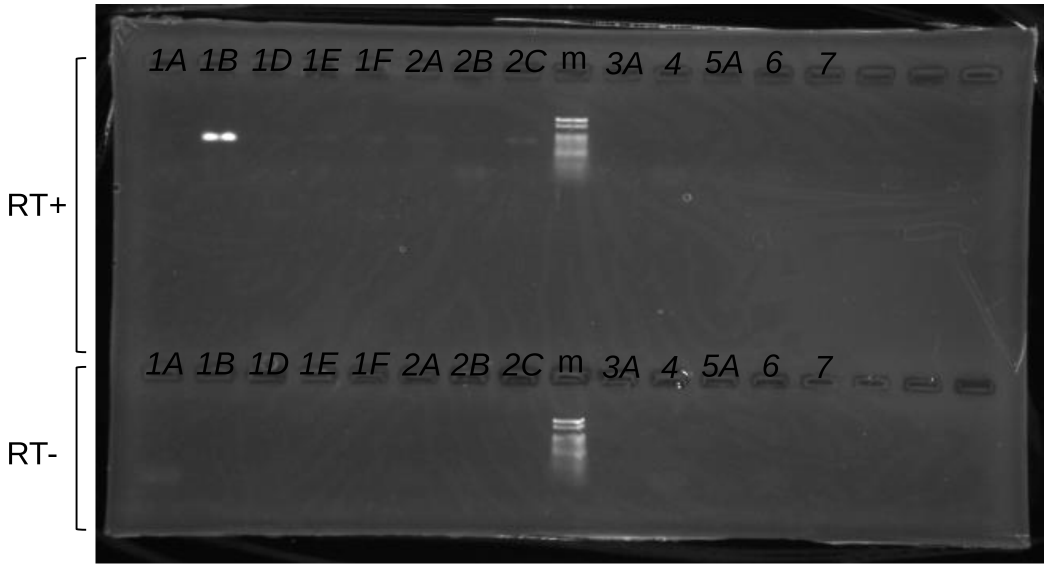

1A: 5-HTR1A; 1B: 5-HTR1B; 1D: 5-HTR1D; 1E: 5-HTR1E; 1F: 5-HTR1F; 2A: 5-HTR2A; 2B: 5-HTR2B; 2C: 5-HTR2C; 3A: 5-HTR3A; 4: 5-HTR4; 5A: 5-HTR5A; 7: 5-HTR7; bp: base pair; m: molecular marker; RT: reverse-transcription

Supplemental Figure 4. Dopaminergic nuclei positions detected by *TH*-expression in the chick midbrains

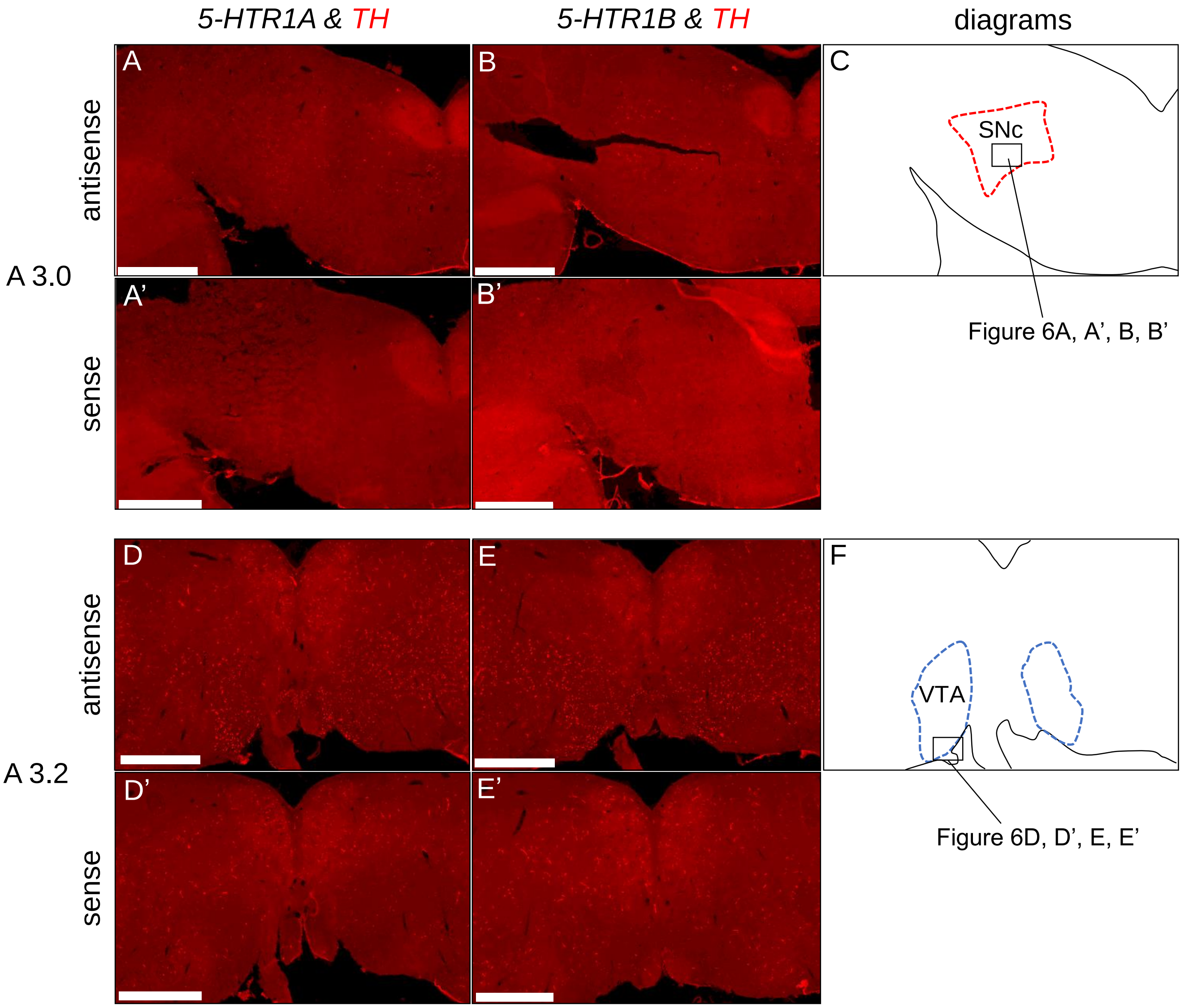

Supplement: Supplementary file 1 [file DataSheet1.PDF]
